# Whole genome sequencing of Turkish genomes reveals functional private alleles and impact of genetic interactions with Europe, Asia and Africa

**DOI:** 10.1186/1471-2164-15-963

**Published:** 2014-11-07

**Authors:** Can Alkan, Pinar Kavak, Mehmet Somel, Omer Gokcumen, Serkan Ugurlu, Ceren Saygi, Elif Dal, Kuyas Bugra, Tunga Güngör, S Cenk Sahinalp, Nesrin Özören, Cemalettin Bekpen

**Affiliations:** Department of Genome Sciences, University of Washington, Seattle, WA 98195 USA; Department of Computer Engineering, Bilkent University, Ankara, 06800 Turkey; Department of Computer Engineering, Boğaziçi University, İstanbul, 34342 Turkey; TÜBİTAK - BİLGEM - UEKAE (The Scientific and Technological Research Council of Turkey, Informatics and Information Security Research Center, National Research Institute of Electronics and Cryptology), Gebze, Kocaeli 41470 Turkey; Department of Integrative Biology, University of California, Berkeley, CA 94720 USA; Department of Biology, Middle East Technical University, Ankara, 06800 Turkey; Department of Biological Sciences, University at Buffalo, Buffalo, NY 14260 USA; Department of Molecular Biology and Genetics, Boğaziçi University, İstanbul, 34342 Turkey; School of Computing Science, Simon Fraser University, Burnaby, BC V5A 1S6 Canada; Max-Planck Institute for Evolutionary Biology, August-Thienemannstrasse 2, Plön, 24306 Germany

## Abstract

**Background:**

Turkey is a crossroads of major population movements throughout history and has been a hotspot of cultural interactions. Several studies have investigated the complex population history of Turkey through a limited set of genetic markers. However, to date, there have been no studies to assess the genetic variation at the whole genome level using whole genome sequencing. Here, we present whole genome sequences of 16 Turkish individuals resequenced at high coverage (32 × -48×).

**Results:**

We show that the genetic variation of the contemporary Turkish population clusters with South European populations, as expected, but also shows signatures of relatively recent contribution from ancestral East Asian populations. In addition, we document a significant enrichment of non-synonymous private alleles, consistent with recent observations in European populations. A number of variants associated with skin color and total cholesterol levels show frequency differentiation between the Turkish populations and European populations. Furthermore, we have analyzed the 17q21.31 inversion polymorphism region (*MAPT* locus) and found increased allele frequency of 31.25% for H1/H2 inversion polymorphism when compared to European populations that show about 25% of allele frequency.

**Conclusion:**

This study provides the first map of common genetic variation from 16 western Asian individuals and thus helps fill an important geographical gap in analyzing natural human variation and human migration. Our data will help develop population-specific experimental designs for studies investigating disease associations and demographic history in Turkey.

**Electronic supplementary material:**

The online version of this article (doi:10.1186/1471-2164-15-963) contains supplementary material, which is available to authorized users.

## Background

High throughput sequencing technologies have prompted sequencing of human genomes at the population level. For instance, the 1000 Genomes Project has reported genome resequencing data from 14 populations and aims to analyze the genomes of a total of 27 populations [1, 2]. These projects provide us with immense amounts of information regarding human genomic variation and the functional properties of such variation. Still, the coverage of world-wide variation remains limited, and virtually no whole genome resequencing data is available involving populations in western Asia, a region encompassing the eastern Mediterranean basin and the Middle East [3].

Western Asia has been the main corridor through which initial out-of-Africa migrations have populated Eurasia [4]. Moreover, the region experienced a massive demographic expansion during the early Holocene with the establishment of large, agricultural societies in the region [5], which subsequently shaped the genetic structure of Europe through migrations [6]. The demography of the region was later influenced by the movement of Turkic speaking populations beginning from 11^th^ Century A.D., which occurred with disputed intensity and frequency, but with undoubtedly profound linguistic impact [7]. In addition to early population movements, there were subsequent population events that had a major influence on the genetic variation in the region, including demographic shrinkage and early 20th Century migration, followed by major population expansion and local migrations in the late 20th and early 21st Century [8]. Locus specific population genetic studies have addressed some of these issues, demarcating Y chromosome [9, 10], mtDNA [11], *Alu* [12] and SNP genotypes [13] in the general Turkish population. However, an integrative, genome-wide assessment of genomic variation is still missing.

In addition to providing insights into the demographic history of the population, resequencing studies are gaining prominence for identifying variants that are associated with complex, sometimes population specific diseases [14]. Such assessments are not possible using array technologies, as array probes are designed based on common variation and hence suffer from ascertainment bias [15]. It is expected that most common genomic variation is shared among populations and only a small percentage of overall genomic variation is confined to geographic regions. However, rare or private (i.e., those that are confined to a particular population) variants have stirred recent attention and may explain heritable diseases and local adaptation [16–18]. Such variants may explain the genetic components of diseases that occur at unusual frequencies in Turkey, including Behçet’s [19], familial Mediterranean fever [20], and beta thalassemia [21].

In this paper, we present, for the first time, high coverage (32X to 48X) whole genome re-sequencing data from 16 individuals from Turkey, covering at least 99.1% of the genome at 1X, and at least 98.79% at 5X (Table 1, Additional file 1: Table S1). We sampled the genomes from diverse geographical regions in Turkey, leading to the identification of 651,936 novel SNVs, 542,508 novel indels, and a non-redundant total of 10,731 deletion polymorphisms. It has been shown previously in comparable population genomics studies that even extremely high accuracy SNP calling pipelines cannot avoid a small fraction of false-positive SNPs. These have been shown to be significantly enriched among population specific and rare variants. For instance, a recent study has predicted to have approximately 2,000 false-positive SNPs per genome, even if the overall SNP calling accuracy is 99.4% and the majority of these SNPs were found to be population specific [22]. To assess the extend to which the population specific variants we detected are false-positives, we used PCR followed by Sanger sequencing. Indeed, of the 24 variable SNV and indel sites that are population specific 7 of them (~29%) were falsely called as variable sites. This is in contrast with our overall validation results and indicates much higher false-positive rate among the novel variants. However, these results do not change our observation that there are hundreds of thousands of novel variants found in the Turkish population.

**Table 1:** **Summary of the Turkish genome project**

Sample ID	Coverage	SNPs	Novel SNPs	Indels ^1^	Novel indels	Deletions ^2^	Novel deletions ^3^
06A010111	36.45	3,238,983	40,780	915,917	223,334	1,939	245
08P210611	36.58	3,258,882	45,582	904,093	216,377	1,690	176
24D220611	39.68	3,274,222	46,468	912,202	219,227	1,711	202
25A220611	33.37	3,241,675	46,364	905,503	217,648	1,676	188
31P140611	36.72	3,238,064	47,316	903,106	217,024	1,718	198
32A140611	33.56	3,268,102	42,525	907,291	217,036	1,726	192
33M140611	32.41	3,255,966	43,745	902,853	214,684	1,743	197
34S291210	37.66	3,251,620	42,144	914,379	221,851	1,881	223
35C240511	34.42	3,241,914	39,450	910,516	219,466	1,735	184
38I220611	35.44	3,231,738	46,475	902,232	216,872	1,681	183
42S291210	31.33	3,254,639	44,041	887,958	208,584	1,669	168
48S210611	38.17	3,302,283	43,599	914,063	218,293	1,700	178
50G301210	37.80	3,276,506	43,416	920,203	223,146	1,930	249
52C130611	32.63	3,269,131	44,621	916,643	219,997	1,888	240
57M220611	31.60	3,213,229	42,705	891,337	212,644	1,654	182
65A220611	48.09	3,259,571	48,211	915,187	222,548	1,676	184
Non redundant Total		8,161,894	647,131	1,729,238	526,177	3,292	494

^1^Indels between 1–50 bp. ^2^Deletions >50 bp. ^3^Deletions that are not previously reported in the 1000 Genomes Project (both 2010 and 2012 releases).

Our results showed that genetic variation within Turkey clusters with European populations, while showing signatures of admixture from African and East Asian populations, consistent with influence of potential North African interactions and Altaic admixture. Based on our analysis of SNPs reported in GWAS studies that show the highest frequency differences between Turkey and European populations, we find SNPs associated with pigmentation and cholesterol level.

## Results and discussion

### Whole genome analysis and variant discovery

**Figure 1:**
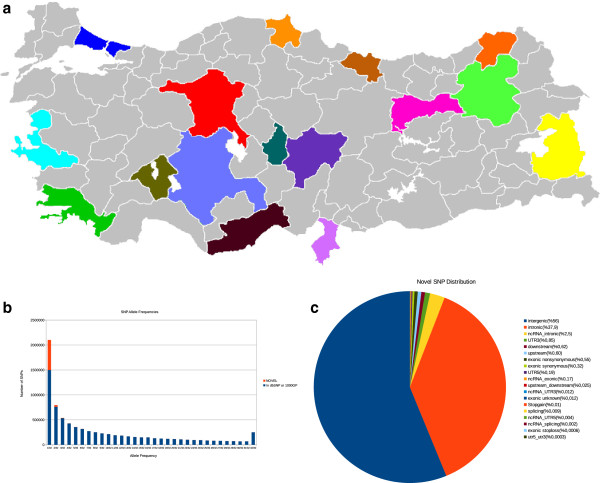
**Brief summary of the Turkish Genome Project. (A)** A map of Turkey showing provinces where volunteers were recruited in color. **(B)** Allele frequencies of the SNPs found in Turkish samples and annotated as novel vs. known (dbSNP135 + 1000 Genomes Project). **(C)** Functional annotation of the novel SNPs.

We recruited 16 healthy volunteers from across Turkey (Figure 1A). The individuals were included in the study irrespective of their mother-tongue/ethnicity; we refer to them collectively as “Turkish”.

We isolated DNA from blood samples obtained from each individual, and generated whole genome shotgun (WGS) sequence data at high coverage ranging between 32 to 48X, using the Illumina platform (Methods, Table 1, Additional file 1: Table S1). Using BWA [23] and GATK [24], we identified an average of 3,254,782 SNPs per individual, and a non-redundant total of 8,161,894 SNPs, of which 651,936 (7.99%) were found to be novel when compared to dbSNP v132 and the 1000 Genomes Project (Figure 1B). We observed that 3,583 of the novel SNPs found were identified as non-synonymous mutations in protein coding sequence (Figure 1C). We also indentified a total of 1,765,584 short (<50 bp) indels. Additionally, using VariationHunter [25] we identified 1,751 large (50 bp to 100 Kbp) deletions per individual on average (Table 1).

To estimate the false discovery and false negative rates (FDR and FNR) of our SNP calls, we analyzed the same samples using Affymetrix 6.0 SNP arrays (Methods) to genotype for common SNPs. We then compared the microarray results with the SNP calls we generated from WGS. Assuming the SNP arrays as the gold standard, we estimated the FDR to be at 0.174% and FNR as 0.209%.

### Common genetic variation in contemporary Turkey

Next we studied the 16 genomes with respect to major patterns of population diversity, structure, and migration, comparing these profiles with those in the 1000 Genomes Project [2]. We found that genome-wide nucleotide diversity (π) in Turkey is comparable to that observed in Europeans, while lower than in Africans or admixed Native American populations, and higher than in East Asians (Additional file 2: Figure S1). Even though we expect lower values of π in Turkish populations as compared to African populations, it was surprising to find π in European populations equal to or slightly higher than that observed in Turkey. We expected that Turkish genomes might exhibit significantly higher nucleotide diversity, given Turkey’s location at the crossroads of out-of-Africa migrations, as well as more recent population movements [9]. Still, this result should be taken with caution given differences in SNP calling procedures and power between the Turkish and 1000 Genomes Project datasets, which could potentially affect estimated diversity levels.

**Figure 2:**
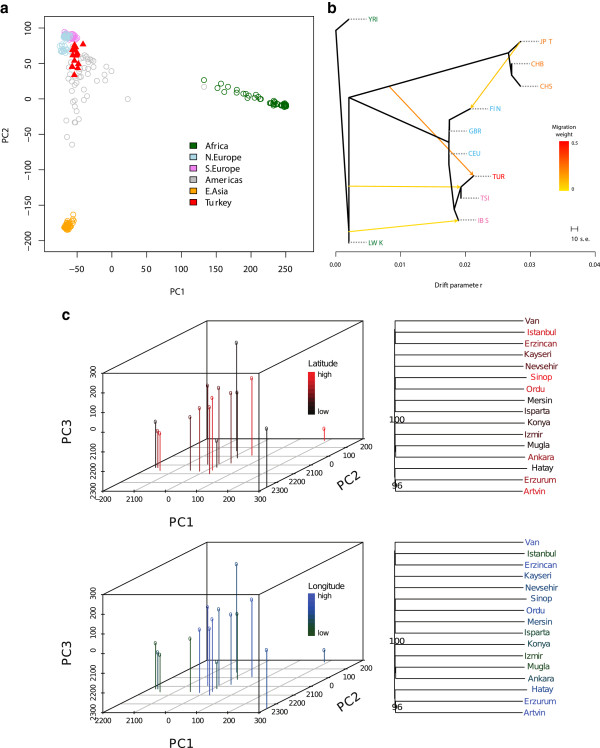
**Population genetic relationships between Turkey and world-wide populations. (A)** The first two principal components of the Turkish genome dataset combined with 16 individual population subsets from the 1000 Genomes Project dataset. The first and second components explain 6% and 5% of the total variance, respectively. **(B)** A population tree based on “Treemix” analysis. The populations included are as follows: Turkey (TUR); Toscani in Italia (TSI); Iberian populations in Spain (IBS); British from England and Scotland (GBR); Finnish from Finland (FIN); Utah residents with Northern and Western European ancestry (CEU); Han Chinese in Beijing, China (CHB); Japanese in Tokyo, Japan (JPT); Han Chinese South (CHS); Yoruba in Ibadan, Nigeria (YRI); Luhya in Webuye, Kenya (LWK). Populations with high degree of admixture (Native American and African American populations) were not included to simplify the analysis. The Yoruban population was used to root the tree. In total four migration events were estimated. The weights for the migration events predicted to originate from the East Asian branch into current-day Turkey was 0.217, from the ancestral Eurasian branch into the Turkey-Tuscan clade was 0.048, from the African branch into Iberia was 0.026, from the Japanese branch into Finland was 0.079. **(C)** The first three principal components of the Turkish genome dataset (left panels) and neighbor-joining trees of the 16 subjects (right panels). The upper and lower panels show the same data, except for being colored according to latitude and longitude of subject locations, respectively. The first, second and third principal components each explain ~7% of the total variance. Names of the provinces where each sample are recruited from are listed in Additional file 1: Table S1.

To obtain an overview of population relationships, we conducted principal component analyses using 16 individuals randomly selected from each one of the 14 populations within the 1000 Genomes Project, combined with the profiles from Turkey (Methods). The first two principal components of the combined dataset replicated the global genetic structure identified in earlier studies (e.g. [26]). Within this picture, the genetic variation observed among Turkish population clusters with variation observed in European populations (Figure 2A). The genome profiles from Turkey also overlapped with those of Native American populations. This is interesting, because the genetic affinity of Native American populations to both East Asia and Europe has traditionally been attributed to their Asian origins and subsequent admixture with Europeans [27]. However, this picture has recently been reinterpreted as evidence of shared Ancient North Eurasian ancestry in Native Americans and Europeans [28]. This result implies that significant Ancient North Eurasian ancestry might also be found in Turkish genetic profiles; this requires further study.

We then estimated the maximum likelihood population tree with migration using the Treemix software [29] (Methods). The Treemix program models populations as having ancestry from multiple parental populations, it can then calculate migration weights [29]. These weights are correlated with the fraction of alleles contributed by a parental population; however, they are not an unbiased predictor of this fraction.

In the Treemix analysis, Turkish samples clustered together with South Europe/Mediterranean populations: Iberians from Spain and Tuscans from Italy (Figure 2B). Within the worldwide population tree, two of the four predicted migration events involved Turkey. The strongest predicted migration event represents admixture from the root of the East Asian branch into Turkey, which could be reflecting Central/South Asian population migration [10]. The second migration pattern represents admixture from the root of the Eurasian branch (close to the African branches) to the common node between Turkey and Italy. The algorithm predicts a parallel branch from Africa into Spain. These patterns plausibly reflect South Mediterranean admixture into North Mediterranean, as observed for other populations in the Mediterranean basin [30].

The weight for the migration event predicted to originate from the branch ancestral to all Eurasians (presumably Middle East and North Africa), to the Turkey-Tuscan clade, was only 0.048. In comparison, the weight for the migration event predicted to originate from the branch ancestral to East Asia (presumably Central Asia) into current-day Turkey was 0.217. Although this implies a major population event from the East to West Asia, we note that these weights are not direct estimates of the migration rates. First, the original contributing populations to the ancestral population in Turkey are not known. For instance, we do not know the exact genetic relationship between current-day East Asian populations and the Turkic speakers from Central Asia who migrated into Anatolia about 1,000 years before present. In fact, Hodoglugil and Mahley, using HGDP genotyping data, predict that South Asian contribution to Turkey's population was significantly higher than East/Central Asian contributions [13], suggesting that the genetic variation of medieval Central Asian populations may be more closely related to South Asian populations, or that there was continued low level migration from South Asia into Anatolia. Another possibility is Ancient North Eurasian genetic contribution to both the historical Anatolian and East Asian populations [28], which might have been interpreted as migration in this dataset. Second, Pickrell and Pritchard [29] also note that in their simulations, the weights underestimate relatively high admixture proportions. Data from more closely related populations coupled with extensive population genetic simulation may eventually allow determining the relative contributions of migration events that shaped population variation in Turkey.

Finally, we investigated possible population structure within Turkey. Principal component analyses did not reveal any subclustering among the 16 individuals, and we found no evidence for longitudinal or latitudinal divergence (Figure 2C). Supporting this lack of structure, we found no correlation between genetic distances among subjects and geographic distances among subject locations (Mantel test p > 0.10). One explanation for this observed pattern is the well-documented high-levels of recent population mobility within this geographic region [31, 32]. However, this pattern might not be exclusive to Turkey; for example, a neighbor joining tree of 98 Tuscan individuals in the 1000 Genomes Project similarly displays a star-like phylogeny (Additional file 3: Figure S2), i.e., we observe long terminal branches coalescing at about the same time, indicating lack of structure within the population.

### Genetic variants exhibiting unusual frequency in the Turkish samples

**Figure 3:**
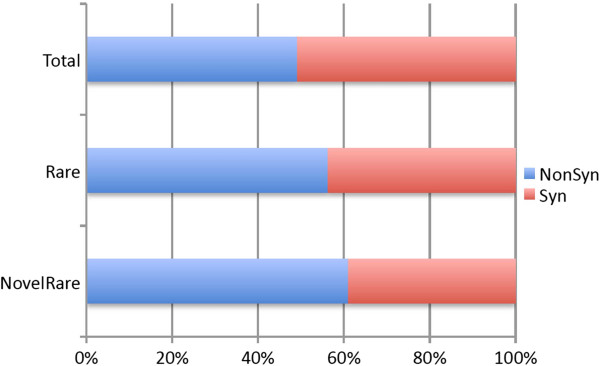
**The proportion of synonymous and non-synonymous SNPs.** SNPs that are found in a single chromosome among the 16 Turkish genomes (Rare) show a significant increase in the proportion of non-synonymous SNPs (NonSyn) to synonymous SNPs (Syn), as compared to all SNPs observed in the same population (p < 0.01, Chi-square with Yates’ correction). This increase is even more visible among SNPs that are seen in a single chromosome and are novel (i.e., not found in other databases) (NovelRare).

The assessment of whole genome and exome sequencing data at the population level has shown that due to recent population growth, human populations carry an excess of rare genetic variants [17], many of which can be functional [33]. Indeed, we identified 5,523 synonymous or non-synonymous rare –i.e., variants that were found in a single Turkish chromosome but not in any other population - and 7 synonymous or non-synonymous private –found in more than 3 Turkish chromosomes, but not in any other population- single nucleotide variants (SNVs) in the Turkish population (Additional file 4: Table S2). As expected, rare SNVs are significantly more likely to be non-synonymous than SNPs observed in multiple populations (Figure 3). We further scrutinized 3 non-synonymous private alleles. Interestingly, one of these genes, *CCDC82*, was shown to evolve under positive selection in humans, and diverged from Neandertals [34]. Indeed, the non-synonymous private SNP in the Turkish population observed at chr19:50832152, is a T- > C mutation and homologous to Denisovan haplotype at the orthologous site.

**Figure 4:**
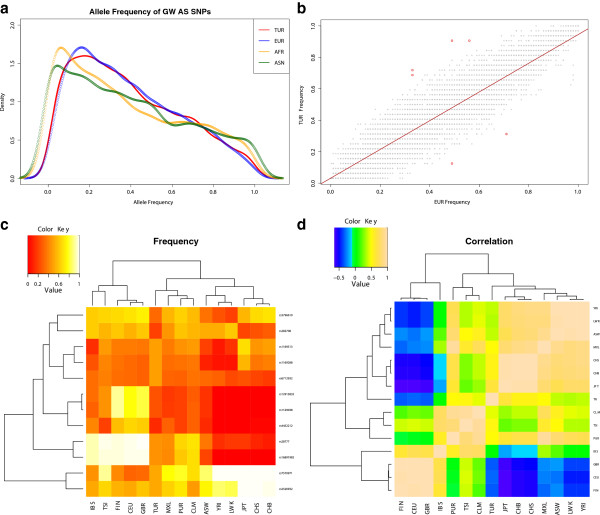
**The allele frequency of GWAS SNPs. (A)** The density distribution of allele frequency of GWAS SNPs among world populations. The distribution of GWAS SNPs in Turkey (TUR) is more similar to that of the European population (EUR) as compared to East Asian (ASN) and African (AFR) populations, indicating a greater proportion of ancestry sharing between TUR and EUR. Meanwhile, there is a higher proportion of common SNPs in both TUR and EUR than ASN and AFR, which is likely due to ascertainment bias in the GWAS studies as described in [35]. **(B)** The allele frequency of GWAS SNPs between Turkish (y-axis) and European (x-axis) populations. The red dots indicate SNPs in the 0.1 percentile (>0.345 allele frequency difference) of the absolute allele frequency distributions between Turkish and European populations. rs1129038 and rs12913832, both affecting HERC2 gene, are in strong linkage disequilibrium and, as such, have identical allele frequencies in European and Turkish populations. They are represented as two overlapping dots with 0.31 allele frequency in Turkish population (y-axis) and 0.71 allele frequencies in European population (x-axis). **(C)** The frequency distribution of derived and ancestral alleles for rs6712932 in Africa (ASW, LWK, YRI) Europe (CEU, FIN, GBR, IBS, TSI), Asia (CHB, CHS, JPT) and Americas (CLM, MXL, PUR) and Turkey (TUR). Note the increased frequency of the derived allele in the Turkish population.

We then explored potentially functional variants at higher or lower frequency in the Turkish population relative to the closely related European populations (Figure 4). For this, we compiled the frequencies of published GWAS SNPs among samples resequenced by the 1000 Genomes Project [2]. We then compared the frequencies of these SNPs in continental populations with what we observed in the Turkish population (Figure 4A). As expected, the frequency distribution of SNPs in the Turkish population is, by and large, similar to that observed among European populations. To identify potential outliers to this expectation, we compared the frequency distributions of the European and Turkish populations and identified 7 SNPs displaying the highest frequency differences between these populations (Figure 4B, 0.1st percentile, corresponding to > ~0.35 absolute difference in frequencies).

Among these 7 SNPs, 2 exist at lower frequencies in Turkey compared to Europe. We found that both are related to pigmentation [36], and hair color [37]. Pigmentation is a genetic and variable trait in humans [38], with lighter skin color being associated with better vitamin D absorption, but higher incidence of skin cancer [39]. As such, human pigmentation correlates with latitude and the low frequency of this SNP in Turkish population as compared to Europe may reflect positive selection for these alleles in Northern Europe and/or selection against the alleles in Southern Europe and Turkey.

To our surprise, 2 of the 5 SNPs that show higher frequency in Turkish population as compared to Europeans are associated with lower total cholesterol counts. This is particularly interesting, given that the Turkish population has indeed been shown to have lower cholesterol than Western European populations, including total cholesterol, and high and low density lipoprotein cholesterol measures, while carrying relatively high triglyceride levels [40]. Although cholesterol levels are strongly shaped by diet and lifestyle, they are also under the influence of genetic factors [41]. The frequency distribution of one of these SNPs (rs7570971), associated with total cholesterol counts [42], is fixed in Asian continent and almost fixed among African populations, decreased to slightly above 90% in Turkish populations, further decreasing to 49% among Europeans. Therefore, similar to what is observed for pigmentation related SNPs, rs7570971 is best explained by clinal adaptation or drift patterns. More striking is the frequency distribution of rs1169288, another SNP associated with total cholesterol count [42], which reaches almost 70% in Turkish populations, but is at lower than 10% frequency in Africa and remains a minor allele in other Eurasian populations (except Japan, where it reaches 54%) (Figure 4C). Intriguingly, however, for both cholesterol level related SNPs, the variant at high frequency in Turkey (A for rs7570971 and C for rs1169288) is reported to elevate total cholesterol levels [42]. Hence, the contribution of these variants to phenotypic differences between populations needs further study.

### Deletion polymorphisms

We used the mrFAST aligner [43] together with the VariationHunter [44] algorithm to discover deletions in the genomes of the samples resequenced in this study. We merged individual sample-based callsets, then used the Genome STRiP tool [45] to both genotype and *in silico* validate our discovery callset (Methods). In summary, we identified 1,751 deletions per individual, with a non-redundant total of 3,292 deletions. To assess novelty, we compared against the 1000 Genomes Project datasets (Methods), and found 199 novel deletions per sample on average (494 total). We did not find any whole-gene deletions, but 21/494 deletions were predicted to delete coding exons where 10/21 were predicted in more than one chromosome (allele frequency >3%; Additional file 5: Table S3).

We also analyzed the genomic structure at the 17q21 locus, a region with increased plasticity [46–49], where eight different haplotypes are characterized [46]. Previous studies have shown that all eight haplotypes show themselves as an inversion polymorphism that exist as two main variants: a direction-orientation haplotype, H1, prevalent in most human populations and an inverted haplotype, H2 which predominantly occurs in European populations [46–48] and presents itself as an 970-kbp inversion [49]. This complex region has been shown to be associated with increased fecundity and an increase in global recombination and it was shown that the H2 haplotype is enriched in Europeans [46, 49]. We have found an allele frequency of 31.25% for 17q21.31 H1/H2 inversion polymorphism in the samples we analyzed, which shows an increase when compared to European populations at 25% allele frequency [48]. One of the samples, sequenced at 34X coverage, was also homozygous for H2 inversion allele (Additional file 1: Table S1 and Additional file 6: Figure S3).

## Conclusion

Although the 1000 Genomes Project published in 2012 [2] had aimed to provide a comprehensive map of human genetic variation, it was not complete: populations in the Eastern Mediterranean and the Middle East were missing from that study. In this paper, we present data from high depth whole genome sequencing of 16 individuals from modern day Turkey to complement the 1000 Genomes Project in an effort to extend our understanding of normal human genetic variation. We provide the first preliminary genome-wide map of single nucleotide variation, as well as deletion polymorphisms in this population and in western Asia.

Our analyses show that genetic variation of the contemporary Turkish population is best described within the context of the Southern European/Mediterranean gene pool. However, we predict notable genetic sharing between Turkey’s population and East Asian and African populations. As expected from recent studies, rare and private genetic variation in Turkey has presumably more functional impact than variation shared among populations. We further identified SNPs that were previously associated with diseases that show allele frequency differentiation between Turkey and other Western European populations. Among these, those associated with pigmentation were at lower frequencies in Turkey than in Europe; meanwhile variants associated with total cholesterol levels were at higher levels in the former. Overall, our study improves the framework for population genomics studies in the region, and should incite novel genome-wide association studies in Turkey. Future studies using larger sample sizes will be able to elucidate population structure and history in more detail.

## Methods

### Ethics statement and sample collection

Institutional review board permission was obtained from INAREK (Committee on Ethical Conduct in Studies Involving Human Subjects at the Boğaziçi University). We collected blood samples from 16 volunteers after receiving signed informed consent forms from each of the individuals. The individuals that participated in this study were selected to represent different geographical regions of Turkey.

### DNA extraction from human blood sample – NaCl extraction

Approximately 10 ml peripheral blood was collected from each subject into a tube containing K3EDTA to prevent coagulation. Each blood sample was then transferred into a 50 ml centrifuge tube and 30 ml of ice cold red blood cell (RBC) lysis buffer was added. The contents were mixed thoroughly and the mixture was kept at 4°C for at least 20 minutes to lyse the cell membranes. Centrifugation was performed at 5000 rpm at 4°C for 10 minutes. The supernatant containing the RBC debris was removed and the pellet containing the leukocyte nuclei was washed with 3 ml RBC lysis buffer to remove the cell debris. The pellet was suspended in 10 ml of cold RBC lysis buffer by vortexing. After centrifugation at 5000 rpm at 4°C for 10 minutes, the supernatant was discarded, the pellet was cleaned with 3 ml RBC lysis buffer and centrifuged again. 3 ml nuclei lysis buffer was added and the pellet was dissolved by vortexing. In order to digest nuclear proteins, 30 ul Proteinase K (20 mg/ml) and 50 ul of 10% SDS were added, mixed gently and incubated at 37°C overnight or at 56°C for 3 hours. After the incubation step, 10 ml 2.5 M NaCl solution was added to the mixture, shaken thoroughly and centrifuged at 5000 rpm at 20°C for 30 minutes. The supernatant was transferred into a new 50 ml centrifuge tube and approximately 30 ml absolute ethanol was added. The tube was inverted gently until the DNA threads become visible. DNA was fished out with the aid of a micropipette and was left to dry for 2 hours. It was dissolved in 300 ul TE and stored at -20°C. The purity and quality of the extracted DNA were measured at A260/A280, and were found to be within the acceptable range (1.8 - 2.0).

### Sequencing

Whole genome shotgun sequence data were generated using the Illumina HiSeq2000 platform (paired-end 101 bp reads clipped to 90 bp) at BGI-Shenzhen. The sequencing reads that contained Illumina adapter sequences or high number of low-quality bases were removed. We finally obtained sequence data coverage between 32- to 48-fold per sample.

### SNP and indel discovery

We aligned the reads to the human reference genome (NCBI GRCh37) using the BWA aligner [23] with default options and paired-end mode (*sampe*). For easier comparison and mitochondrial DNA correspondence, we used the GRCh37 version that is used by the 1000 Genomes Project. Next we used the GATK tool [24] to realign indel-containing reads to the reference genome. We then used GATK UnifiedGenotyper in multi-sample mode to generate SNP and indel callsets separately by pooling the data from 16 genomes. We applied the Variant Quality Score Recalibration filter using the GATK resource bundle version 1.2 to help minimize false positives. We then removed any SNP and indel calls that overlap with segmental duplications to help further reduce false positives. We used the experimental validation data (described below) to approximately calculate the sensitivity and specificity of these call sets.

### Validation with SNP microarrays

We hybridized the DNA extracted from 15 of the samples with the Affymetrix 6.0 SNP arrays to test for sensitivity and specificity of the call sets. We compared the Affymetrix 6.0 SNP array results with SNPs we obtained from WGS. Assuming that microarray results provide the gold standard, we calculated false discovery rate to be 0.174% on average and false negative rate to be 0.209%.

### Deletion polymorphism discovery and validation

Using the BAM files generated in the previous step, we filtered read pairs that map concordantly to the reference genome within 4 standard deviations around the mean span size. In order to discover all possible mapping locations of the remaining discordant read pairs and unmapped reads in GRCh37, we used the mrFAST aligner [43] in paired-end mode allowing edit distance of at most 4 for both reads. Next, we used the VariationHunter algorithm [44] to call putative deletions (>50 bp) less than 100 Kbp. To calculate the non-redundant set of deletions, we pairwise merged any deletion calls that overlapped at least 80% reciprocally. We then used the GenomeSTRiP tool [45] to genotype the deletions discovered by VariationHunter. We also used the GenomeSTRiP results as a surrogate for *in silico* validation, and we filtered out any calls that were not successfully genotyped using split-read or read-depth information. We reasoned that any *true* variation discovered with VariationHunter using read-pair information will be supported by read-depth and split-read information used by GenomeSTRiP to genotype the variant in the genomes of multiple individuals. Although it is still possible that GenomeSTRiP may have false negatives in genotyping, therefore incorrectly invalidating a true variant detected by VariationHunter, this effect is acceptable to obtain a more reliable (lower FDR) call set.

As a second *in silico* validation strategy, we counted the number of heterozygous SNPs detected by GATK within the novel deletion intervals. We applied this validation to only the novel deletion calls when compared to the 1000 Genomes Project. We reasoned that, if a deletion is real, then it couldn’t contain heterozygous SNPs within, as at least one of the copies is deleted. The only exception to this rule is if the deletion is found within duplication. However, such deletions are too difficult to detect to start with, and filtered out by both VariationHunter and GenomeSTRiP. We found that >85% of our novel deletion calls contain no heterozygous SNPs; where only 10% contains exactly one heterozygous SNP.

We determined novelty in our call set by filtering those deletions that overlap (>50% reciprocal) with any of the 1000 Genomes Project releases [1, 2], or calls discovered within the 1000 Genomes Project but not genotyped. We used the liftOver tool to convert the NCBI Build36 coordinates to GRCh37 for the 1000 Genomes Project Pilot Project results [1].

### PCR validation of the 17q21.31 haplotypes

Genotypes of each individual were confirmed by PCR by using the primer specific for Tau deletion region using primers 5’GGAAGACGTTCTCACTGATCTG, and 3’AAGAGTCTGGCTTCAGTCTCTC and subsequent capillary based sequence analysis [49, 50].

### Variant annotation

We used ANNOVAR (version 2012Oct23) to annotate the detected variants. For gene based and filter based annotation, we used the April 2012 version of the annotation database (hg19-1000g2012apr_all, hg19_ALL.sites.2012_04.txt), and the dbSNP version 132. For comparison against the 1000 Genomes Project, we used the data released by the consortium as Phase I of the project [2].

### Population genetic analyses

To compare Turkish populations with worldwide populations, we used the phase 1 version of the 1000 Genomes Project dataset [2]. Average nucleotide diversity was calculated in each dataset separately, using biallelic autosomal SNPs passing the respective quality thresholds. The sum of π values across all SNPs was divided by sum of ungapped chromosome lengths (GRCh37). Next, to compare populations in the two datasets directly, we combined the datasets. For simplicity, as well as to avoid technical biases, we restricted our analysis to biallelic autosomal SNPs polymorphic in both datasets, totaling 7,134,695. We pruned this dataset to remove SNPs in high linkage using the PLINK software, using the recommended parameters (window size = 50, shift = 5, r^2 = 0.5) [51], which yielded 1,090,112 SNPs. To further limit ascertainment bias, we randomly chose 16 individual subsets (the same number as in the Turkish sample) from each of the 14 populations represented in 1000 Genomes Project, and required a SNP to be polymorphic in each of these 14 population subsets (except for the IBS population, which originally had 14 samples). This step further reduced the number of SNPs to 208,816. Genotypes were coded as follows: 1 for homozygous reference, 2 for heterozygous, and 3 for homozygous alternative. Using this dataset we first conducted principal component (PC) analyses using the ‘prcomp’ function in the R environment, after scaling the matrix to unit variance. To further resolve relationships among populations, we used a recently developed method, *Treemix*, that allows constructing maximum likelihood phylogenetic trees while allowing migration from edges to nodes [29], providing an efficient alternative to STRUCTURE [52] and similar programs for studying population structure. We removed populations with known large admixture (Native American and African American populations) to focus on admixture events in Turkey. We ran the program with Yorubans as root, grouping SNPs in bins of 100, and allowing for four migrations. We further performed principal component analyses and constructed a neighbor joining tree (using the ‘nj’ function in the R ‘ape’ package) on the 16 individuals from Turkey. We additionally calculated the matrix of genetic distances between individuals and compared these distances with geographic distances calculated from longitude and latitude of subject locations, using the Mantel test.

### GWAS analysis

To compare the frequencies of SNPs associated with phenotypes and disease between Turkish population and other world populations, we calculated the frequencies of SNPs listed in the GWAS Catalog [53] among world populations using data from 1000 Genomes Project [2]. Then, we have conducted a pairwise comparison of frequencies of these SNPs observed in Turkish population with populations included in 1000 Genomes Project (Additional file 7: Figure S4). Our initial results, concordant with our population genetics analysis, indicated that the frequency distribution of GWAS SNPs in European populations is closest to that observed in the Turkish populations. As such, we have calculated the distribution of absolute frequency differences between Turkish and European populations and identified the outliers (0.1 percentile) (Figure 4).

### Availability of supporting data

Sequence reads are deposited to the SRA read archive (SRP021510). The VCF file that lists all genomic variation characterized in this study is available at: http://turkiyegenomprojesi.boun.edu.tr/tgp_vcf/TGP.integrated_callset.vcf.gz.

## Electronic supplementary material

Additional file 1: Table S1: Geographic locations of the samples used in the project and their NCBI accession numbers. (PDF 81 KB)

Additional file 2: Figure S1: Average nucleotide diversity (π) across the genome calculated for the 1000 Genomes dataset populations and the 16 Turkish genomes. Positions with missing data were removed. Hardy-Weinberg filtering was not applied. The populations included are as follows: Turkey (TUR); Utah residents with Northern and Western European ancestry (CEU); Toscani in Italia (TSI); British from England and Scotland (GBR); Finnish from Finland (FIN); Iberian populations in Spain (IBS); Han Chinese in Beijing, China (CHB); Japanese in Tokyo, Japan (JPT); Han Chinese South (CHS); Yoruba in Ibadan, Nigeria (YRI); Luhya in Webuye, Kenya (LWK); African Ancestry in Southwest US (ASW); Mexican Ancestry in Los Angeles, CA (MXL); Puerto Rican in Puerto Rico (PUR); Colombian in Medellin, Colombia (CLM). (PDF 454 KB)

Additional file 3: Figure S2: Neighbor joining tree of Tuscan individuals from Italy from the 1000 Genomes Project. Individuals are indexed according to their order in the 1000 Genomes dataset. Note the star-like topology of the tree. (PDF 78 KB)

Additional file 4: Table S2: Characterization of the 7 private SNPs. (PDF 97 KB)

Additional file 5: Table S3: Novel deletions discovered in the TGP dataset. (PDF 2 MB)

Additional file 6: Figure S3: To validate our prediction for common inversion polymorphism, we have selected three individuals that are shown to be polymorphic for H1/H1 (38I220611), H1/H2 (33 M140611) and H2/H2 (32A140611) haplotypes. **A)** Read alignments for three individuals within MAPT deletion region (238 bp) [49, 50] are given. **B)** Genotypes of each individual are also confirmed by RT-PCR and subsequent Sanger-based sequencing PCR analysis are performed to confirm the genotype of H1 and H2 individuals using a diagnostic indel [50]. (PDF 348 KB)

Additional file 7: Figure S4: Pairwise comparison of GWAS SNP allele frequencies. Note the expected high correlation of allele frequencies between Turkish and European populations. (PDF 266 KB)

## References

[1] 1000 Genomes Project (2010). A map of human genome variation from population-scale sequencing. Nature.

[2] 1000 Genomes Project (2012). An integrated map of genetic variation from 1,092 human genomes. Nature.

[3] Ozcelik T, Kanaan M, Avraham KB, Yannoukakos D, Megarbane A, Tadmouri GO, Middleton L, Romeo G, King MC, Levy-Lahad E (2010). Collaborative genomics for human health and cooperation in the Mediterranean region. Nat Genet.

[4] Gignoux CR, Henn BM, Mountain JL (2011). Rapid, global demographic expansions after the origins of agriculture. Proc Natl Acad Sci U S A.

[5] Bellwood PS (2005). First Farmers : the origins of agricultural societies.

[6] Semino O, Magri C, Benuzzi G, Lin AA, Al-Zahery N, Battaglia V, Maccioni L, Triantaphyllidis C, Shen P, Oefner PJ, Zhivotovsky LA, King R, Torroni A, Cavalli-Sforza LL, Underhill PA, Santachiara-Benerecetti AS (2004). Origin, diffusion, and differentiation of Y-chromosome haplogroups E and J: inferences on the neolithization of Europe and later migratory events in the Mediterranean area. Am J Hum Genet.

[7] Cahen C (1968). Pre-Ottoman Turkey; a general survey of the material and spiritual culture and history, c. 1071–1330.

[8] Clark B (2006). Twice a stranger : the mass expulsions that forged modern Greece and Turkey.

[9] Cinnioglu C, King R, Kivisild T, Kalfoglu E, Atasoy S, Cavalleri GL, Lillie AS, Roseman CC, Lin AA, Prince K, Oefner PJ, Shen P, Semino O, Cavalli-Sforza LL, Underhill PA (2004). Excavating Y-chromosome haplotype strata in Anatolia. Hum Genet.

[10] Di Benedetto G, Erguven A, Stenico M, Castri L, Bertorelle G, Togan I, Barbujani G (2001). DNA diversity and population admixture in Anatolia. Am J Phys Anthropol.

[11] Calafell F, Underhill P, Tolun A, Angelicheva D, Kalaydjieva L (1996). From Asia to Europe: mitochondrial DNA sequence variability in Bulgarians and Turks. Ann Hum Genet.

[12] Berkman CC, Dinc H, Sekeryapan C, Togan I (2008). Alu insertion polymorphisms and an assessment of the genetic contribution of Central Asia to Anatolia with respect to the Balkans. Am J Phys Anthropol.

[13] Hodoglugil U, Mahley RW (2012). Turkish population structure and genetic ancestry reveal relatedness among Eurasian populations. Ann Hum Genet.

[14] Lupski JR, Belmont JW, Boerwinkle E, Gibbs RA (2011). Clan genomics and the complex architecture of human disease. Cell.

[15] Clark AG, Hubisz MJ, Bustamante CD, Williamson SH, Nielsen R (2005). Ascertainment bias in studies of human genome-wide polymorphism. Genome Res.

[16] Tennessen JA, Bigham AW, O'Connor TD, Fu W, Kenny EE, Gravel S, McGee S, Do R, Liu X, Jun G, Kang HM, Jordan D, Leal SM, Gabriel S, Rieder MJ, Abecasis G, Altshuler D, Nickerson DA, Boerwinkle E, Sunyaev S, Bustamante CD, Bamshad MJ, Akey JM, Broad GO, Seattle GO, NHLBI Exome Sequencing Project (2012). Evolution and functional impact of rare coding variation from deep sequencing of human exomes. Science (New York, NY).

[17] Keinan A, Clark AG (2012). Recent explosive human population growth has resulted in an excess of rare genetic variants. Science (New York, NY).

[18] Fu W, O'Connor TD, Jun G, Kang HM, Abecasis G, Leal SM, Gabriel S, Altshuler D, Shendure J, Nickerson DA, Bamshad MJ, Akey JM, NHLBI Exome Sequencing Project (2013). Analysis of 6,515 exomes reveals the recent origin of most human protein-coding variants. Nature.

[19] Remmers EF, Cosan F, Kirino Y, Ombrello MJ, Abaci N, Satorius C, Le JM, Yang B, Korman BD, Cakiris A, Aglar O, Emrence Z, Azakli H, Ustek D, Tugal-Tutkun I, Akman-Demir G, Chen W, Amos CI, Dizon MB, Kose AA, Azizlerli G, Erer B, Brand OJ, Kaklamani VG, Kaklamanis P, Ben-Chetrit E, Stanford M, Fortune F, Ghabra M, Ollier WE, Cho YH, Bang D, O'Shea J, Wallace GR, Gadina M, Kastner DL, Gül A (2010). Genome-wide association study identifies variants in the MHC class I, IL10, and IL23R-IL12RB2 regions associated with Behcet's disease. Nat Genet.

[20] Dundar M, Emirogullari EF, Kiraz A, Taheri S, Baskol M (2011). Common Familial Mediterranean Fever gene mutations in a Turkish cohort. Mol Biol Rep.

[21] Tadmouri GO, Garguier N, Demont J, Perrin P, Basak AN (2001). History and origin of beta-thalassemia in Turkey: sequence haplotype diversity of beta-globin genes. Hum Biol.

[22] Ju YS, Kim JI, Kim S, Hong D, Park H, Shin JY, Lee S, Lee WC, Kim S, Yu SB, Park SS, Seo SH, Yun JY, Kim HJ, Lee DS, Yavartanoo M, Kang HP, Gokcumen O, Govindaraju DR, Jung JH, Chong H, Yang KS, Kim H, Lee C, Seo JS (2011). Extensive genomic and transcriptional diversity identified through massively parallel DNA and RNA sequencing of eighteen Korean individuals. Nat Genet.

[23] Li H, Durbin R (2009). Fast and accurate short read alignment with Burrows-Wheeler transform. Bioinformatics (Oxford, England).

[24] Depristo MA, Banks E, Poplin R, Garimella KV, Maguire JR, Hartl C, Philippakis AA, Del Angel G, Rivas MA, Hanna M, McKenna A, Fennell TJ, Kernytsky AM, Sivachenko AY, Cibulskis K, Gabriel SB, Altshuler D, Daly MJ (2011). A framework for variation discovery and genotyping using next-generation DNA sequencing data. Nat Genet.

[25] Hormozdiari F, Alkan C, Eichler EE, Sahinalp SC (2009). Combinatorial algorithms for structural variation detection in high-throughput sequenced genomes. Genome Res.

[26] Li JZ, Absher DM, Tang H, Southwick AM, Casto AM, Ramachandran S, Cann HM, Barsh GS, Feldman M, Cavalli-Sforza LL, Myers RM (2008). Worldwide human relationships inferred from genome-wide patterns of variation. Science (New York, NY).

[27] Bryc K, Velez C, Karafet T, Moreno-Estrada A, Reynolds A, Auton A, Hammer M, Bustamante CD, Ostrer H (2010). Colloquium paper: genome-wide patterns of population structure and admixture among Hispanic/Latino populations. Proc Natl Acad Sci U S A.

[28] Lazaridis I, Patterson N, Mittnik A, Renaud G, Mallick S, Kirsanow K, Sudmant PH, Schraiber JG, Castellano S, Lipson M, Berger B, Economou C, Bollongino R, Fu Q, Bos KI, Nordenfelt S, Li H, de Filippo C, Prüfer K, Sawyer S, Posth C, Haak W, Hallgren F, Fornander E, Rohland N, Delsate D, Francken M, Guinet JM, Wahl J, Ayodo G (2014). Ancient human genomes suggest three ancestral populations for present-day Europeans. Nature.

[29] Pickrell JK, Pritchard JK (2012). Inference of population splits and mixtures from genome-wide allele frequency data. PLoS Genet.

[30] Moorjani P, Patterson N, Hirschhorn JN, Keinan A, Hao L, Atzmon G, Burns E, Ostrer H, Price AL, Reich D (2011). The history of African gene flow into Southern Europeans, Levantines, and Jews. PLoS Genet.

[31] Tekeli I (1990). Osmanli Imparatorlugu'ndan Günümüze Nüfusun Zorunlu Yer Değiştirmesi ve Iskan Sorunu’. Toplum ve Bilim.

[32] Kolluoğlu B (2013). Excesses of nationalism: Greco-Turkish population exchange. Nations and Nationalism.

[33] Nelson MR, Wegmann D, Ehm MG, Kessner D, St Jean P, Verzilli C, Shen J, Tang Z, Bacanu SA, Fraser D, Warren L, Aponte J, Zawistowski M, Liu X, Zhang H, Zhang Y, Li J, Li Y, Li L, Woollard P, Topp S, Hall MD, Nangle K, Wang J, Abecasis G, Cardon LR, Zöllner S, Whittaker JC, Chissoe SL, Novembre J, Mooser V (2012). An abundance of rare functional variants in 202 drug target genes sequenced in 14,002 people. Science (New York, NY).

[34] Crisci JL, Wong A, Good JM, Jensen JD (2011). On characterizing adaptive events unique to modern humans. Genome Biol Evol.

[35] Rosenberg NA, Huang L, Jewett EM, Szpiech ZA, Jankovic I, Boehnke M (2010). Genome-wide association studies in diverse populations. Nat Rev.

[36] Jin Y, Birlea SA, Fain PR, Ferrara TM, Ben S, Riccardi SL, Cole JB, Gowan K, Holland PJ, Bennett DC, Luiten RM, Wolkerstorfer A, van der Veen JP, Hartmann A, Eichner S, Schuler G, van Geel N, Lambert J, Kemp EH, Gawkrodger DJ, Weetman AP, Taïeb A, Jouary T, Ezzedine K, Wallace MR, McCormack WT, Picardo M, Leone G, Overbeck A, Silverberg NB, Spritz RA (2012). Genome-wide association analyses identify 13 new susceptibility loci for generalized vitiligo. Nat Genet.

[37] Han J, Kraft P, Nan H, Guo Q, Chen C, Qureshi A, Hankinson SE, Hu FB, Duffy DL, Zhao ZZ, Martin NG, Montgomery GW, Hayward NK, Thomas G, Hoover RN, Chanock S, Hunter DJ (2008). A genome-wide association study identifies novel alleles associated with hair color and skin pigmentation. PLoS Genet.

[38] Myles S, Somel M, Tang K, Kelso J, Stoneking M (2007). Identifying genes underlying skin pigmentation differences among human populations. Hum Genet.

[39] Jablonski NG, Chaplin G (2010). Colloquium paper: human skin pigmentation as an adaptation to UV radiation. Proc Natl Acad Sci U S A.

[40] Ujcic-Voortman JK, Bos G, Baan CA, Uitenbroek DG, Verhoeff AP, Seidell JC (2010). Ethnic differences in total and HDL cholesterol among Turkish, Moroccan and Dutch ethnic groups living in Amsterdam, the Netherlands. BMC Public Health.

[41] Hegele RA (2009). Plasma lipoproteins: genetic influences and clinical implications. Nat Rev.

[42] Teslovich TM, Musunuru K, Smith AV, Edmondson AC, Stylianou IM, Koseki M, Pirruccello JP, Ripatti S, Chasman DI, Willer CJ, Johansen CT, Fouchier SW, Isaacs A, Peloso GM, Barbalic M, Ricketts SL, Bis JC, Aulchenko YS, Thorleifsson G, Feitosa MF, Chambers J, Orho-Melander M, Melander O, Johnson T, Li X, Guo X, Li M, Shin Cho Y, Jin Go M, Jin Kim Y (2010). Biological, clinical and population relevance of 95 loci for blood lipids. Nature.

[43] Alkan C, Kidd JM, Marques-Bonet T, Aksay G, Antonacci F, Hormozdiari F, Kitzman JO, Baker C, Malig M, Mutlu O, Sahinalp SC, Gibbs RA, Eichler EE (2009). Personalized copy number and segmental duplication maps using next-generation sequencing. Nat Genet.

[44] Hormozdiari F, Hajirasouliha I, Dao P, Hach F, Yorukoglu D, Alkan C, Eichler EE, Sahinalp SC (2010). Next-generation VariationHunter: combinatorial algorithms for transposon insertion discovery. Bioinformatics (Oxford, England).

[45] Handsaker RE, Korn JM, Nemesh J, McCarroll SA (2011). Discovery and genotyping of genome structural polymorphism by sequencing on a population scale. Nat Genet.

[46] Steinberg KM, Antonacci F, Sudmant PH, Kidd JM, Campbell CD, Vives L, Malig M, Scheinfeldt L, Beggs W, Ibrahim M, Lema G, Nyambo TB, Omar SA, Bodo JM, Froment A, Donnelly MP, Kidd KK, Tishkoff SA, Eichler EE (2012). Structural diversity and African origin of the 17q21.31 inversion polymorphism. Nat Genet.

[47] Sharp AJ, Hansen S, Selzer RR, Cheng Z, Regan R, Hurst JA, Stewart H, Price SM, Blair E, Hennekam RC, Fitzpatrick CA, Segraves R, Richmond TA, Guiver C, Albertson DG, Pinkel D, Eis PS, Schwartz S, Knight SJ, Eichler EE (2006). Discovery of previously unidentified genomic disorders from the duplication architecture of the human genome. Nat Genet.

[48] Stefansson H, Helgason A, Thorleifsson G, Steinthorsdottir V, Masson G, Barnard J, Baker A, Jonasdottir A, Ingason A, Gudnadottir VG, Desnica N, Hicks A, Gylfason A, Gudbjartsson DF, Jonsdottir GM, Sainz J, Agnarsson K, Birgisdottir B, Ghosh S, Olafsdottir A, Cazier JB, Kristjansson K, Frigge ML, Thorgeirsson TE, Gulcher JR, Kong A, Stefansson K (2005). A common inversion under selection in Europeans. Nat Genet.

[49] Bekpen C, Tastekin I, Siswara P, Akdis CA, Eichler EE (2012). Primate segmental duplication creates novel promoters for the *LRRC37* gene family within the 17q21.31 inversion polymorphism region. Genome Res.

[50] Evans W, Fung HC, Steele J, Eerola J, Tienari P, Pittman A, Silva R, Myers A, Vrieze FW, Singleton A, Hardy J (2004). The tau H2 haplotype is almost exclusively Caucasian in origin. Neurosci Lett.

[51] Purcell S, Neale B, Todd-Brown K, Thomas L, Ferreira MA, Bender D, Maller J, Sklar P, de Bakker PI, Daly MJ, Sham PC (2007). PLINK: a tool set for whole-genome association and population-based linkage analyses. Am J Hum Genet.

[52] Pritchard JK, Stephens M, Donnelly P (2000). Inference of population structure using multilocus genotype data. Genetics.

[53] **A Catalog of Published Genome-Wide Association Studies** [http://www.genome.gov/gwastudies]

