# Supplementary material for: Whole genome sequencing of Turkish genomes reveals functional private alleles and impact of genetic interactions with Europe, Asia and Africa
Source: BMC Genomics. 2014 Nov 7;15(1):963. doi: 10.1186/1471-2164-15-963 (PMC4236450; doi:10.1186/1471-2164-15-963)
Supplement: Supplementary file 1 — Additional file 1: Table S1: Geographic locations of the samples used in the project and their NCBI accession numbers. (PDF 81 KB) [file 12864_2014_6660_MOESM1_ESM.pdf]

Supplementary Table 1. Geographic locations of the samples used in the project and their NCBI accession numbers.

Sample ID	Gender	BioSample ID	SRA Accession	17q21 Genotype
06A010111	M	SAMN0205357	SRS416175	H1/H2
08P210611	F	SAMN0205357	SRS416176	H1/H1
24D220611	F	SAMN0205357	SRS416177	H1/H1
25A220611	M	SAMN0205357	SRS416178	H1/H2
31P140611	F	SAMN0205357	SRS416179	H1/H2
32A140611	M	SAMN0205358	SRS416180	H1/H1
33M140611	M	SAMN0205358	SRS416181	H1/H1
34S291210	M	SAMN0205358	SRS416182	H1/H1
35C240511	M	SAMN0205358	SRS416183	H2/H2
38I220611	M	SAMN0205358	SRS416184	H1/H2
42S291210	M	SAMN0205358	SRS416185	H1/H1
48S210611	F	SAMN0205358	SRS416186	H1/H2
50G301210	F	SAMN0205358	SRS416187	H1/H2
52C130611	F	SAMN0205358	SRS416188	H1/H2
57M220611	M	SAMN0205358	SRS416189	H1/H2
65A220611	M	SAMN0205359	SRS416190	H1/H1
