# Supplementary figures and images for: Whole genome sequencing of Turkish genomes reveals functional private alleles and impact of genetic interactions with Europe, Asia and Africa
Source: BMC Genomics. 2014 Nov 7;15(1):963. doi: 10.1186/1471-2164-15-963 (PMC4236450; doi:10.1186/1471-2164-15-963)

Supplementary Figure 1

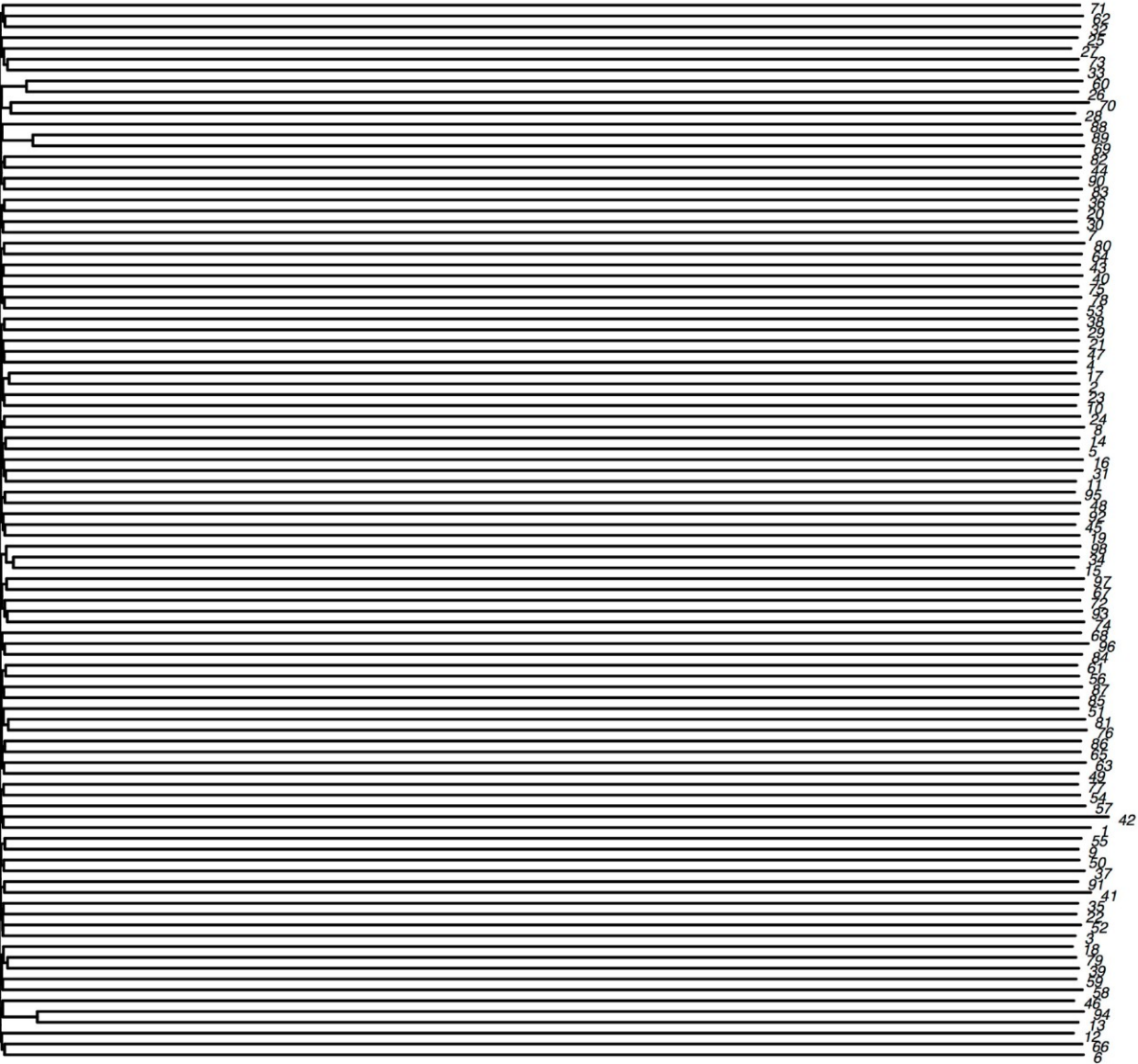

Supplement: Supplementary file 2 — Additional file 2: Figure S1: Average nucleotide diversity (π) across the genome calculated for the 1000 Genomes dataset populations and the 16 Turkish genomes. Positions with missing data were removed. Hardy-Weinberg filtering was not applied. The populations included are as follows: Turkey (TUR); Utah residents with Northern and Western European ancestry (CEU); Toscani in Italia (TSI); British from England and Scotland (GBR); Finnish from Finland (FIN); Iberian populations in Spain (IBS); Han Chinese in Beijing, China (CHB); Japanese in Tokyo, Japan (JPT); Han Chinese South (CHS); Yoruba in Ibadan, Nigeria (YRI); Luhya in Webuye, Kenya (LWK); African Ancestry in Southwest US (ASW); Mexican Ancestry in Los Angeles, CA (MXL); Puerto Rican in Puerto Rico (PUR); Colombian in Medellin, Colombia (CLM). (PDF 454 KB) [file 12864_2014_6660_MOESM2_ESM.pdf]

Supplementary Figure 2

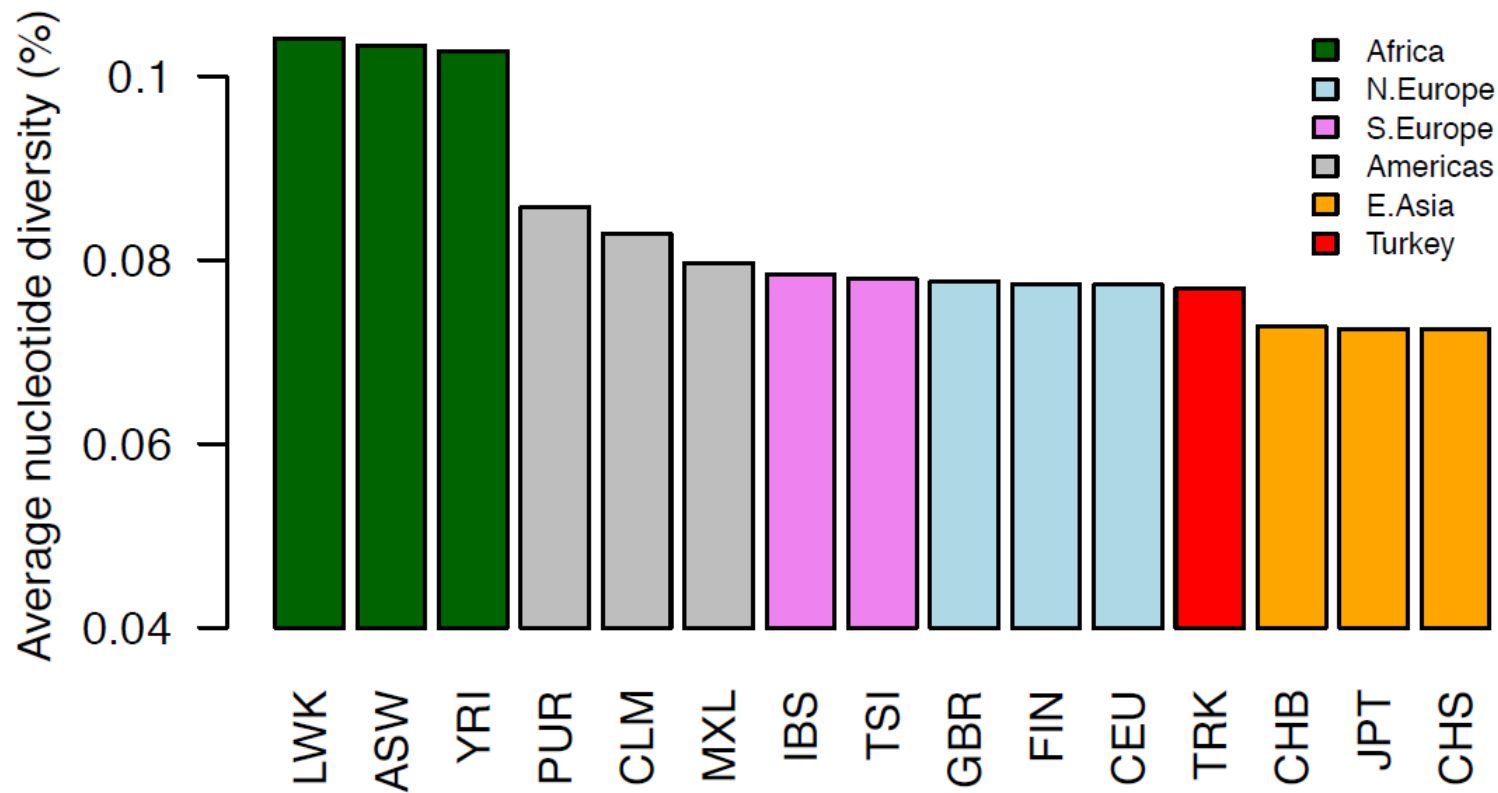

Supplement: Supplementary file 3 — Additional file 3: Figure S2: Neighbor joining tree of Tuscan individuals from Italy from the 1000 Genomes Project. Individuals are indexed according to their order in the 1000 Genomes dataset. Note the star-like topology of the tree. (PDF 78 KB) [file 12864_2014_6660_MOESM3_ESM.pdf]

# Supplementary Figure 3

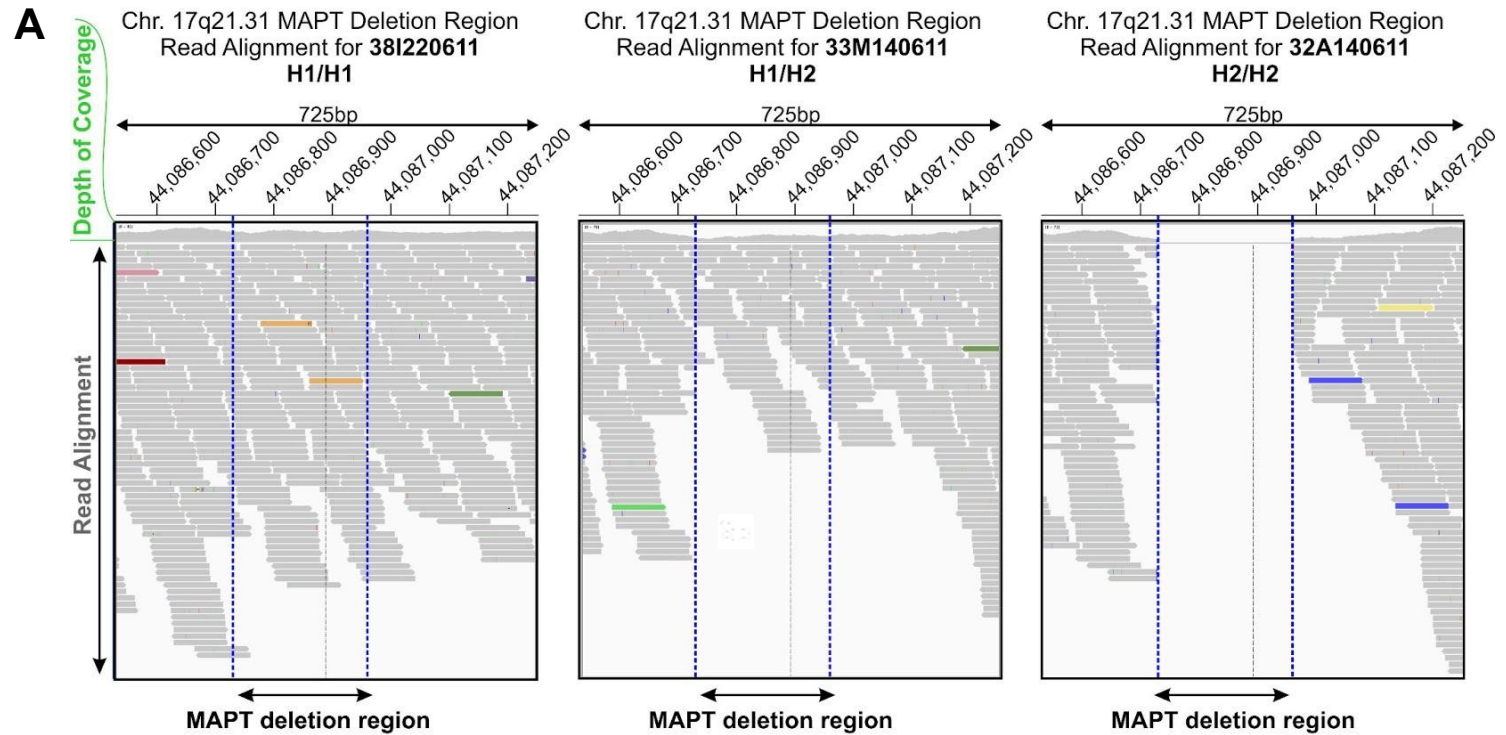

**B**

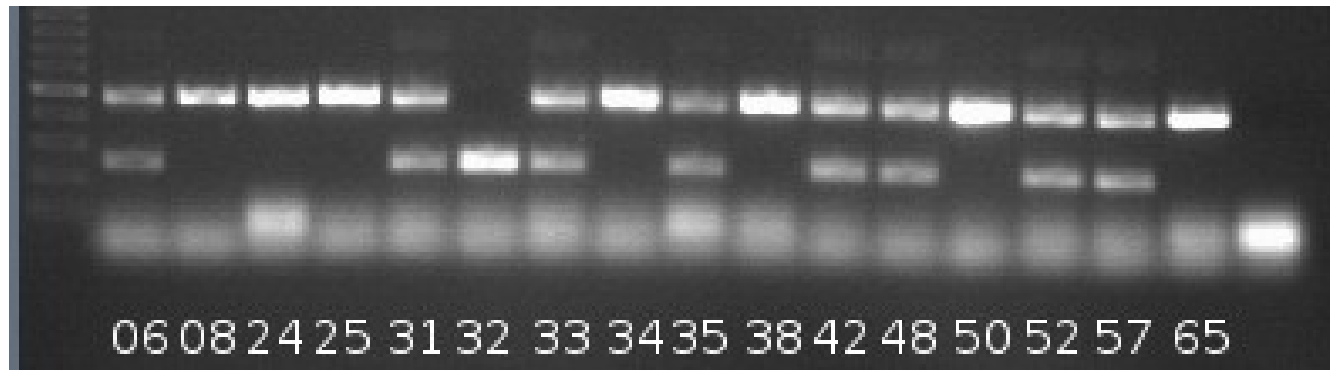

Supplement: Supplementary file 6 — Additional file 6: Figure S3: To validate our prediction for common inversion polymorphism, we have selected three individuals that are shown to be polymorphic for H1/H1 (38I220611), H1/H2 (33 M140611) and H2/H2 (32A140611) haplotypes. A) Read alignments for three individuals within MAPT deletion region (238 bp) [49, 50] are given. B) Genotypes of each individual are also confirmed by RT-PCR and subsequent Sanger-based sequencing PCR analysis are performed to confirm the genotype of H1 and H2 individuals using a diagnostic indel [50]. (PDF 348 KB) [file 12864_2014_6660_MOESM6_ESM.pdf]

# Supplementary Figure 4

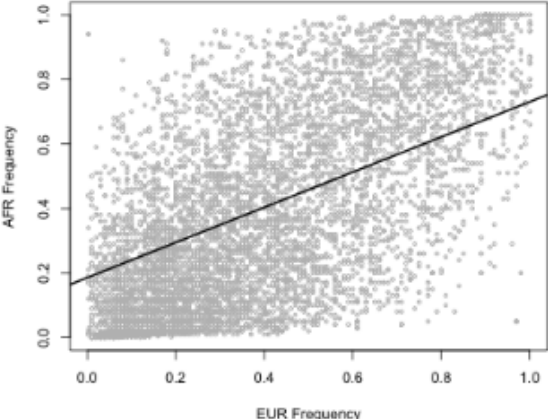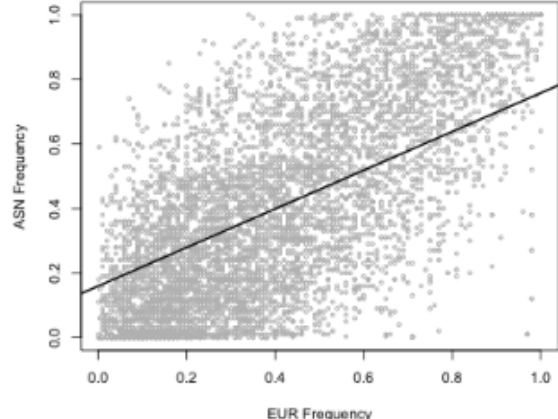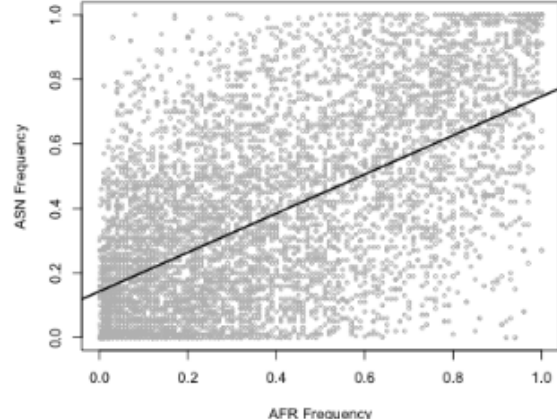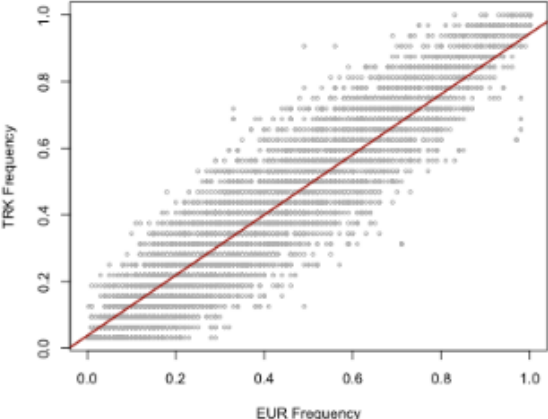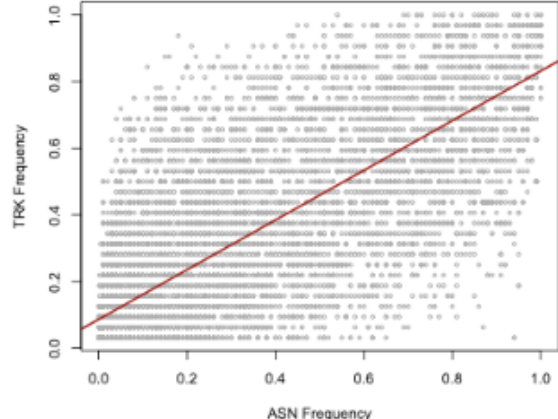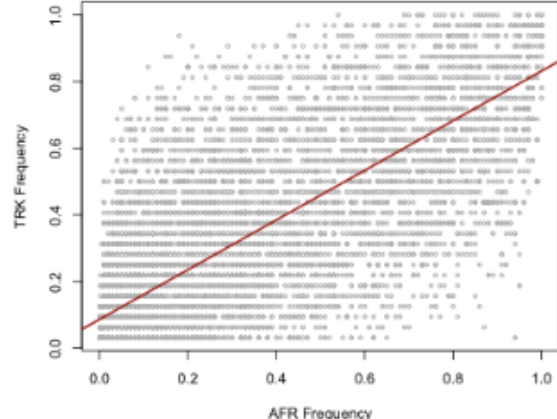

Supplement: Supplementary file 7 — Additional file 7: Figure S4: Pairwise comparison of GWAS SNP allele frequencies. Note the expected high correlation of allele frequencies between Turkish and European populations. (PDF 266 KB) [file 12864_2014_6660_MOESM7_ESM.pdf]
