# Supplementary material for: Whole genome sequencing of Turkish genomes reveals functional private alleles and impact of genetic interactions with Europe, Asia and Africa
Source: BMC Genomics. 2014 Nov 7;15(1):963. doi: 10.1186/1471-2164-15-963 (PMC4236450; doi:10.1186/1471-2164-15-963)
Supplement: Supplementary file 4 — Additional file 4: Table S2: Characterization of the 7 private SNPs. (PDF 97 KB) [file 12864_2014_6660_MOESM4_ESM.pdf]

Supplementary Table 2. Characterization of the 7 private SNPs

Chrom	Position	Ref	Alt	AA_change	Gene_name	Type	100 vertebrates Basewise Conservation by PhyloP	SIFT_score	SIFT_pred	Polyphen-	Polyphen-2_pred
chr1	27689476	A	C	MAP3K6:NM_004672:exon7:c.T1008G:p.S336S	MAP3K6	exonic_synonymous SNV	-1.86625	NA	NA	NA	NA
chr2	1.75E+08	A	G	SP9:NM_001145250:exon2:c.A1386G:p.A462A	SP9	exonic_synonymous SNV	-1.01309	NA	NA	NA	NA
chr4	1222084	G	C	CTBP1:NM_001328:exon3:c.C243G:p.L81L,CTBP1:NM_001012614:exon4:c.C210G:p.L70L	CTBP1	exonic_synonymous SNV	-0.0405512	NA	NA	NA	NA
chr4	88537558	C	T	DSPP:NM_014208:exon5:c.C3744T:p.N1248N	DSPP	exonic_synonymous SNV	-0.159213	NA	NA	NA	NA
chr8	1.45E+08	C	G	EPPK1:NM_031308:exon1:c.G7132C:p.D2378H	EPPK1	exonic_nonsynonymous SNV	7.55143	0.001	damaging	1	probably damaging
chr11	96117520	C	T	CCDC82:NM_024725:exon4:c.G392A:p.S131N	CCDC82	exonic_nonsynonymous SNV	-1.03842	1	tolerated	0.002	benign
chr19	50832152	T	C	KCNC3:NM_004977:exon1:c.A188G:p.D63G	KCNC3	exonic_nonsynonymous SNV	-0.566134	1	tolerated	0	benign

The columns AA_change, Gene_name, dbID, and Type data were obtained from the hg19_ljb_all.txt file of annovar tool. We also used SIFT predictions (http://sift.jcvi.org/, accessed April 2014), pre-calculated Polyphen-2 predictions (http://genetics.bwh.harvard.edu/pph2/dbsearch.shtml, accessed April 2014) and the 100 Vertebrate alignment PhyloP scores (the UCSC Genome Browser hg19, accessed April 2014).
