# Supplementary material for: Whole genome sequencing of Turkish genomes reveals functional private alleles and impact of genetic interactions with Europe, Asia and Africa
Source: BMC Genomics. 2014 Nov 7;15(1):963. doi: 10.1186/1471-2164-15-963 (PMC4236450; doi:10.1186/1471-2164-15-963)
Supplement: Supplementary file 5 — Additional file 5: Table S3: Novel deletions discovered in the TGP dataset. (PDF 2 MB) [file 12864_2014_6660_MOESM5_ESM.pdf]

Supplementary Table 3. Novel deletions discovered in the TGP dataset.

chrom	start	end	length	Allele Freq.	Repeats	Repeats %	SD	SD %	WSSD	WSSD %	Genes (bp)	Coding exons (bp)	Genes
chr1	1,225,117	1,225,535	418	46.88%	394	94.26%	0	0.00%	0	0.00%	418	0	*SCNN1D*
chr1	1,910,391	1,910,614	223	21.88%	0	0.00%	0	0.00%	0	0.00%	223	0	*KIAA1751*
chr1	4,059,848	4,060,129	281	37.50%	0	0.00%	0	0.00%	0	0.00%	0	0	
chr1	8,560,775	8,562,624	1,849	50.00%	1756	94.97%	0	0.00%	0	0.00%	1849	0	*RERE*
chr1	14,711,616	14,712,109	493	50.00%	493	100.00%	0	0.00%	0	0.00%	0	0	
chr1	27,129,892	27,130,300	408	100.00%	408	100.00%	0	0.00%	0	0.00%	0	0	
chr1	30,913,397	30,914,889	1,492	50.00%	1492	100.00%	0	0.00%	0	0.00%	0	0	
chr1	31,474,690	31,474,945	255	3.13%	228	89.41%	0	0.00%	0	0.00%	255	0	*PUM1*
chr1	34,296,434	34,300,370	3,936	50.00%	3936	100.00%	0	0.00%	0	0.00%	3936	0	*CSMD2*
chr1	37,050,224	37,052,050	1,826	3.13%	1059	58.00%	0	0.00%	0	0.00%	0	0	
chr1	39,474,650	39,477,400	2,750	3.13%	2204	80.15%	0	0.00%	0	0.00%	0	0	
chr1	41,024,285	41,024,524	239	12.50%	239	100.00%	0	0.00%	0	0.00%	0	0	
chr1	42,189,825	42,192,228	2,403	3.13%	938	39.03%	0	0.00%	0	0.00%	2403	0	*HIVEP3*
chr1	45,983,451	45,983,734	283	37.50%	39	13.78%	0	0.00%	0	0.00%	283	0	*PRDX1*
chr1	63,064,863	63,065,260	397	3.13%	397	100.00%	0	0.00%	0	0.00%	397	0	*ANGPTL3*
chr1	64,900,966	64,901,595	629	6.25%	153	24.32%	0	0.00%	0	0.00%	0	0	
chr1	73,366,019	73,366,609	590	50.00%	590	100.00%	0	0.00%	0	0.00%	0	0	
chr1	80,590,041	80,591,160	1,119	6.25%	200	17.87%	0	0.00%	0	0.00%	0	0	
chr1	93,020,245	93,020,958	713	3.13%	320	44.88%	0	0.00%	0	0.00%	713	0	*EVI5*
chr1	97,596,214	97,596,477	263	43.75%	247	93.92%	0	0.00%	0	0.00%	263	0	*DPYD*
chr1	106,173,537	106,173,745	208	18.75%	208	100.00%	0	0.00%	0	0.00%	0	0	
chr1	147,124,753	147,125,215	462	50.00%	451	97.62%	0	0.00%	0	0.00%	462	0	*ACP6*
chr1	153,247,211	153,255,588	8,377	3.13%	5898	70.41%	0	0.00%	0	0.00%	0	0	
chr1	161,114,027	161,114,322	295	50.00%	285	96.61%	0	0.00%	0	0.00%	0	0	
chr1	163,849,970	163,852,993	3,023	3.13%	1987	65.73%	0	0.00%	0	0.00%	0	0	
chr1	166,913,315	166,914,619	1,304	3.13%	210	16.10%	0	0.00%	0	0.00%	1304	0	*ILDR2*
chr1	174,202,575	174,203,737	1,162	50.00%	1162	100.00%	0	0.00%	0	0.00%	1162	0	*RABGAP1L*
chr1	179,009,561	179,009,838	277	53.13%	277	100.00%	0	0.00%	0	0.00%	277	0	*FAM20B*
chr1	181,387,115	181,388,556	1,441	3.13%	1162	80.64%	0	0.00%	0	0.00%	0	0	
chr1	183,118,997	183,119,197	200	18.75%	12	6.00%	0	0.00%	0	0.00%	0	0	
chr1	195,432,566	195,433,370	804	3.13%	484	60.20%	0	0.00%	0	0.00%	0	0	
chr1	198,004,720	198,005,130	410	3.13%	410	100.00%	0	0.00%	0	0.00%	0	0	
chr1	200,785,923	200,786,233	310	3.13%	265	85.48%	0	0.00%	0	0.00%	310	0	*CAMSAP1L1*
chr1	205,178,582	205,178,797	215	46.88%	0	0.00%	0	0.00%	0	0.00%	215	0	*DSTYK*
chr1	210,319,381	210,322,738	3,357	3.13%	2398	71.43%	0	0.00%	0	0.00%	3357	0	*SYT14*
chr1	226,375,995	226,384,357	8,362	3.13%	6001	71.77%	0	0.00%	0	0.00%	0	0	
chr1	227,897,507	227,898,786	1,279	50.00%	1113	87.02%	0	0.00%	0	0.00%	0	0	
chr1	231,869,292	231,869,576	284	31.25%	284	100.00%	0	0.00%	0	0.00%	284	0	*DISC1*
chr1	232,366,790	232,367,589	799	37.50%	797	99.75%	0	0.00%	0	0.00%	0	0	
chr1	238,457,228	238,459,157	1,929	3.13%	1793	92.95%	0	0.00%	0	0.00%	0	0	
chr1	245,433,438	245,433,737	299	18.75%	271	90.64%	0	0.00%	0	0.00%	299	0	*KIF26B*
chr1	246,949,097	246,949,418	321	28.13%	0	0.00%	0	0.00%	0	0.00%	0	0	
chr10	463,429	463,841	412	40.63%	0	0.00%	0	0.00%	0	0.00%	412	0	*DIP2C*
chr10	825,431	825,793	362	3.13%	0	0.00%	0	0.00%	0	0.00%	0	0	
chr10	1,452,088	1,452,304	216	6.25%	0	0.00%	0	0.00%	0	0.00%	216	0	*ADARB2*
chr10	7,995,873	7,996,916	1,043	25.00%	0	0.00%	0	0.00%	0	0.00%	1043	0	*TAF3*
chr10	9,515,704	9,516,012	308	3.13%	298	96.75%	0	0.00%	0	0.00%	0	0	
chr10	17,294,074	17,294,463	389	9.38%	302	77.64%	0	0.00%	0	0.00%	0	0	
chr10	44,607,771	44,607,985	214	12.50%	214	100.00%	0	0.00%	0	0.00%	0	0	
chr10	47,083,056	47,083,279	223	18.75%	136	60.99%	223	100.00%	223	100.00%	0	0	
chr10	47,589,610	47,589,950	340	15.63%	340	100.00%	340	100.00%	340	100.00%	0	0	
chr10	68,444,896	68,445,439	543	50.00%	491	90.42%	0	0.00%	0	0.00%	543	0	*CTNNA3*
chr10	87,451,097	87,460,948	9,851	3.13%	6082	61.74%	0	0.00%	0	0.00%	9851	0	*GRID1*
chr10	99,274,388	99,274,683	295	3.13%	0	0.00%	0	0.00%	0	0.00%	295	0	*UBTD1*
chr10	99,912,218	99,912,488	270	56.25%	270	100.00%	0	0.00%	0	0.00%	270	0	*C10orf28*
chr10	125,152,695	125,153,082	387	3.13%	244	63.05%	0	0.00%	0	0.00%	0	0	
chr10	128,295,626	128,296,318	692	6.25%	692	100.00%	0	0.00%	0	0.00%	0	0	
chr10	131,677,569	131,678,152	583	3.13%	0	0.00%	0	0.00%	0	0.00%	583	0	*EBF3*
chr10	131,718,280	131,718,589	309	15.63%	0	0.00%	0	0.00%	0	0.00%	309	0	*EBF3*
chr10	134,165,280	134,165,662	382	28.13%	0	0.00%	0	0.00%	0	0.00%	382	0	*LRRC27*
chr10	134,165,984	134,166,398	414	9.38%	0	0.00%	0	0.00%	0	0.00%	414	13	*LRRC27*
chr10	135,424,327	135,424,574	247	12.50%	247	100.00%	0	0.00%	0	0.00%	0	0	
chr11	411,871	412,888	1,017	46.88%	0	0.00%	0	0.00%	0	0.00%	1017	0	*SIGIRR*
chr11	547,867	551,966	4,099	15.63%	1153	28.13%	0	0.00%	0	0.00%	4099	711	*LRRC56*
chr11	24,723,759	24,726,154	2,395	3.13%	862	35.99%	0	0.00%	0	0.00%	2395	0	*LUZP2*
chr11	41,581,996	41,588,102	6,106	3.13%	3806	62.33%	0	0.00%	0	0.00%	0	0	
chr11	45,112,862	45,121,161	8,299	3.13%	4012	48.34%	0	0.00%	0	0.00%	5598	345	*PRDM11*
chr11	47,914,462	47,914,839	377	50.00%	377	100.00%	0	0.00%	0	0.00%	0	0	
chr11	55,900,182	55,900,458	276	3.13%	0	0.00%	0	0.00%	0	0.00%	0	0	
chr11	68,797,567	68,797,798	231	50.00%	225	97.40%	0	0.00%	0	0.00%	0	0	
chr11	76,445,325	76,445,605	280	50.00%	269	96.07%	0	0.00%	0	0.00%	0	0	
chr11	91,891,212	91,891,574	362	9.38%	362	100.00%	0	0.00%	0	0.00%	0	0	
chr11	96,724,867	96,790,991	66,124	3.13%	40667	61.50%	0	0.00%	0	0.00%	0	0	
chr11	99,468,982	99,469,235	253	50.00%	242	95.65%	0	0.00%	0	0.00%	253	0	*CNTN5*
chr11	103,949,533	103,949,763	230	25.00%	0	0.00%	0	0.00%	0	0.00%	230	0	*PDGFD*
chr11	106,014,470	106,016,171	1,701	3.13%	1701	100.00%	0	0.00%	0	0.00%	0	0	
chr11	107,783,464	107,783,724	260	15.63%	260	100.00%	0	0.00%	0	0.00%	0	0	
chr11	123,443,532	123,443,816	284	12.50%	105	36.97%	0	0.00%	0	0.00%	284	0	*GRAMD1B*
chr11	130,301,409	130,301,633	224	50.00%	0	0.00%	0	0.00%	0	0.00%	0	0	
chr11	131,550,105	131,550,439	334	46.88%	178	53.29%	0	0.00%	0	0.00%	334	0	*NTM*
chr12	227,529	227,818	289	3.13%	0	0.00%	0	0.00%	0	0.00%	289	0	*IQSEC3*
chr12	4,015,491	4,016,987	1,496	6.25%	704	47.06%	0	0.00%	0	0.00%	0	0	
chr12	11,420,457	11,420,770	313	46.88%	0	0.00%	0	0.00%	0	0.00%	313	312	*PRB3*
chr12	26,936,746	26,936,964	218	50.00%	218	100.00%	0	0.00%	0	0.00%	218	0	*ITPR2*
chr12	30,528,179	30,530,449	2,270	3.13%	2270	100.00%	0	0.00%	0	0.00%	0	0	
chr12	38,983,501	38,984,069	568	3.13%	304	53.52%	0	0.00%	0	0.00%	0	0	
chr12	39,860,062	39,860,332	270	18.75%	123	45.56%	0	0.00%	0	0.00%	0	0	
chr12	40,107,083	40,107,364	281	3.13%	247	87.90%	0	0.00%	0	0.00%	281	0	*C12orf40*
chr12	51,871,552	51,872,679	1,127	3.13%	604	53.59%	0	0.00%	0	0.00%	1127	0	*SLC4A8*
chr12	55,473,308	55,475,463	2,155	6.25%	1525	70.77%	0	0.00%	0	0.00%	0	0	

chr12	56,242,323	56,242,994	671	3.13%	271	40.39%	0	0.00%	0	0.00%	0	0	
chr12	72,774,448	72,774,662	214	15.63%	214	100.00%	0	0.00%	0	0.00%	214	0	*TRHDE*
chr12	79,197,458	79,197,773	315	21.88%	0	0.00%	0	0.00%	0	0.00%	0	0	
chr12	91,035,142	91,035,396	254	6.25%	236	92.91%	0	0.00%	0	0.00%	0	0	
chr12	123,689,278	123,689,930	652	3.13%	495	75.92%	0	0.00%	0	0.00%	652	0	*MPHOSPH9*
chr12	131,555,629	131,555,879	250	9.38%	227	90.80%	0	0.00%	0	0.00%	250	0	*GPR133*
chr12	133,352,787	133,353,185	398	6.25%	0	0.00%	0	0.00%	0	0.00%	398	0	*GOLGA3*
chr13	23,675,717	23,676,004	287	3.13%	0	0.00%	0	0.00%	0	0.00%	0	0	
chr13	26,837,407	26,838,023	616	50.00%	588	95.45%	0	0.00%	0	0.00%	616	0	*CDK8*
chr13	28,446,306	28,446,690	384	50.00%	384	100.00%	0	0.00%	0	0.00%	0	0	
chr13	30,844,108	30,844,404	296	46.88%	296	100.00%	0	0.00%	0	0.00%	296	0	*KATNAL1*
chr13	30,844,113	30,844,568	455	46.88%	455	100.00%	0	0.00%	0	0.00%	455	0	*KATNAL1*
chr13	45,317,318	45,317,749	431	3.13%	181	42.00%	0	0.00%	0	0.00%	0	0	
chr13	55,634,125	55,634,559	434	34.38%	434	100.00%	0	0.00%	0	0.00%	0	0	
chr13	61,470,137	61,470,834	697	3.13%	194	27.83%	0	0.00%	0	0.00%	0	0	
chr13	84,084,667	84,085,015	348	3.13%	0	0.00%	0	0.00%	0	0.00%	0	0	
chr13	86,652,591	86,655,568	2,977	9.38%	1334	44.81%	0	0.00%	0	0.00%	0	0	
chr13	94,691,754	94,692,381	627	3.13%	0	0.00%	0	0.00%	0	0.00%	627	0	*GPC6*
chr13	111,329,420	111,329,673	253	21.88%	0	0.00%	0	0.00%	0	0.00%	253	30	*CARS2*
chr13	113,330,748	113,330,992	244	18.75%	138	56.56%	0	0.00%	0	0.00%	244	0	*C13orf35*
chr13	113,495,463	113,495,821	358	12.50%	0	0.00%	0	0.00%	0	0.00%	358	0	*ATP11A*
chr13	113,763,092	113,763,536	444	50.00%	0	0.00%	0	0.00%	0	0.00%	444	0	*F7*
chr13	113,886,512	113,886,879	367	9.38%	244	66.49%	0	0.00%	0	0.00%	367	0	*CUL4A*
chr13	114,773,373	114,774,043	670	25.00%	0	0.00%	0	0.00%	0	0.00%	670	0	*RASA3*
chr14	21,895,411	21,895,702	291	46.88%	0	0.00%	0	0.00%	0	0.00%	291	0	*CHD8*
chr14	41,122,300	41,122,518	218	21.88%	218	100.00%	0	0.00%	0	0.00%	0	0	
chr14	42,160,260	42,166,048	5,788	3.13%	5705	98.57%	0	0.00%	0	0.00%	5788	0	*LRFN5*
chr14	56,153,743	56,174,674	20,931	3.13%	10927	52.20%	0	0.00%	0	0.00%	0	0	
chr14	60,239,247	60,244,293	5,046	3.13%	917	18.17%	0	0.00%	0	0.00%	5046	0	*RTN1*
chr14	77,736,003	77,736,222	219	50.00%	214	97.72%	0	0.00%	0	0.00%	219	0	*NGB*
chr14	95,889,564	95,889,936	372	50.00%	4	1.08%	0	0.00%	0	0.00%	372	0	*C14orf49*
chr14	96,622,517	96,622,803	286	50.00%	258	90.21%	0	0.00%	0	0.00%	0	0	
chr14	100,174,582	100,174,898	316	3.13%	316	100.00%	0	0.00%	0	0.00%	316	0	*CYP46A1*
chr14	104,564,220	104,564,504	284	3.13%	0	0.00%	0	0.00%	0	0.00%	284	0	*ASPG*
chr14	104,896,286	104,896,694	408	43.75%	0	0.00%	0	0.00%	0	0.00%	0	0	
chr15	42,081,616	42,086,391	4,775	3.13%	1644	34.43%	0	0.00%	0	0.00%	4775	0	*MAPKBP1*
chr15	42,212,783	42,222,824	10,041	3.13%	4389	43.71%	0	0.00%	0	0.00%	10041	0	*EHD4*
chr15	54,865,670	54,869,421	3,751	3.13%	778	20.74%	0	0.00%	0	0.00%	3751	0	*UNC13C*
chr15	77,355,752	77,357,922	2,170	3.13%	433	19.95%	0	0.00%	0	0.00%	2170	0	*TSPAN3*
chr15	95,824,044	95,826,880	2,836	3.13%	234	8.25%	0	0.00%	0	0.00%	2836	0	*LOC400456*
chr15	101,289,789	101,291,012	1,223	3.13%	1223	100.00%	0	0.00%	0	0.00%	0	0	
chr16	4,105,984	4,109,998	4,014	3.13%	2476	61.68%	0	0.00%	0	0.00%	4014	0	*ADCY9*
chr16	8,197,260	8,203,160	5,900	3.13%	4805	81.44%	0	0.00%	0	0.00%	0	0	
chr16	10,383,443	10,404,600	21,157	3.13%	16409	77.56%	0	0.00%	0	0.00%	0	0	
chr16	46,391,530	46,392,510	980	46.88%	945	96.43%	980	100.00%	980	100.00%	0	0	
chr16	46,404,651	46,406,427	1,776	50.00%	1684	94.82%	1776	100.00%	1776	100.00%	0	0	
chr16	54,164,047	54,164,374	327	46.88%	308	94.19%	0	0.00%	0	0.00%	0	0	
chr16	58,644,520	58,647,721	3,201	3.13%	1865	58.26%	0	0.00%	0	0.00%	3201	0	*CNOT1*
chr16	62,380,387	62,380,916	529	6.25%	284	53.69%	0	0.00%	0	0.00%	0	0	
chr16	82,788,267	82,788,701	434	3.13%	0	0.00%	0	0.00%	0	0.00%	434	0	*CDH13*
chr16	84,482,346	84,484,347	2,001	3.13%	87	4.35%	0	0.00%	0	0.00%	2001	0	*ATP2C2*
chr16	85,150,876	85,154,537	3,661	3.13%	2314	63.21%	0	0.00%	0	0.00%	0	0	
chr16	88,317,429	88,317,667	238	37.50%	0	0.00%	0	0.00%	0	0.00%	0	0	
chr16	88,553,036	88,553,289	253	3.13%	0	0.00%	0	0.00%	0	0.00%	253	0	*ZFPM1*
chr16	89,203,743	89,204,015	272	6.25%	0	0.00%	0	0.00%	0	0.00%	272	0	*ACSF3*
chr16	89,741,861	89,745,405	3,544	50.00%	3510	99.04%	0	0.00%	0	0.00%	0	0	
chr17	149,619	150,037	418	50.00%	0	0.00%	0	0.00%	0	0.00%	418	0	*RPH3AL*
chr17	665,727	665,949	222	12.50%	0	0.00%	0	0.00%	0	0.00%	222	0	*GLOD4*
chr17	1,056,557	1,057,044	487	25.00%	0	0.00%	0	0.00%	0	0.00%	487	0	*ABR*
chr17	1,240,275	1,244,474	4,199	3.13%	3563	84.85%	0	0.00%	0	0.00%	0	0	
chr17	1,412,086	1,412,413	327	31.25%	0	0.00%	0	0.00%	0	0.00%	327	0	*INPP5K*
chr17	2,311,675	2,313,663	1,988	50.00%	1575	79.23%	0	0.00%	0	0.00%	1988	0	*LOC284009*
chr17	3,016,259	3,016,535	276	3.13%	276	100.00%	0	0.00%	0	0.00%	0	0	
chr17	5,685,030	5,685,274	244	46.88%	142	58.20%	0	0.00%	0	0.00%	0	0	
chr17	6,980,614	6,980,855	241	28.13%	138	57.26%	0	0.00%	0	0.00%	241	0	*CLEC10A*
chr17	8,384,435	8,384,733	298	37.50%	0	0.00%	0	0.00%	0	0.00%	298	89	*MYH10*
chr17	9,199,125	9,199,341	216	9.38%	216	100.00%	0	0.00%	0	0.00%	216	0	*STX8*
chr17	9,204,260	9,204,946	686	50.00%	686	100.00%	0	0.00%	0	0.00%	686	0	*STX8*
chr17	10,703,433	10,704,541	1,108	3.13%	587	52.98%	0	0.00%	0	0.00%	1108	0	*LOC100289255*
chr17	18,241,197	18,241,606	409	12.50%	409	100.00%	0	0.00%	0	0.00%	409	0	*SHMT1*
chr17	33,472,773	33,509,354	36,581	3.13%	20412	55.80%	0	0.00%	0	0.00%	34519	2476	*UNC45B*
chr17	35,233,632	35,240,391	6,759	3.13%	3054	45.18%	0	0.00%	0	0.00%	0	0	
chr17	36,975,492	36,975,705	213	9.38%	210	98.59%	0	0.00%	0	0.00%	213	0	*CWC25*
chr17	39,317,206	39,318,092	886	53.13%	262	29.57%	0	0.00%	0	0.00%	0	0	
chr17	48,553,492	48,553,711	219	9.38%	219	100.00%	0	0.00%	0	0.00%	0	0	
chr17	53,908,720	53,909,948	1,228	3.13%	777	63.27%	0	0.00%	0	0.00%	0	0	
chr17	64,794,523	64,795,822	1,299	53.13%	1256	96.69%	0	0.00%	0	0.00%	1299	0	*PRKCA*
chr17	65,398,921	65,408,102	9,181	3.13%	6406	69.77%	0	0.00%	0	0.00%	9181	0	*PITPNC1*
chr17	68,920,166	68,920,372	206	50.00%	188	91.26%	0	0.00%	0	0.00%	0	0	
chr17	77,795,201	77,799,942	4,741	3.13%	2880	60.75%	0	0.00%	0	0.00%	0	0	
chr17	78,329,934	78,330,819	885	3.13%	263	29.72%	0	0.00%	0	0.00%	885	0	*LOC100294362*
chr17	78,463,079	78,463,742	663	46.88%	663	100.00%	0	0.00%	0	0.00%	0	0	
chr17	81,024,618	81,024,885	267	25.00%	0	0.00%	0	0.00%	0	0.00%	0	0	
chr18	5,839,583	5,839,864	281	3.13%	0	0.00%	0	0.00%	0	0.00%	0	0	
chr18	9,444,054	9,444,782	728	3.13%	326	44.78%	0	0.00%	0	0.00%	0	0	
chr18	25,921,841	25,948,098	26,257	3.13%	11953	45.52%	0	0.00%	0	0.00%	0	0	
chr18	26,274,577	26,274,883	306	37.50%	304	99.35%	0	0.00%	0	0.00%	0	0	
chr18	33,162,801	33,163,905	1,104	3.13%	635	57.52%	0	0.00%	0	0.00%	0	0	
chr18	34,207,381	34,210,359	2,978	3.13%	978	32.84%	0	0.00%	0	0.00%	2978	0	*FHOD3*
chr18	51,831,678	51,832,046	368	34.38%	368	100.00%	0	0.00%	0	0.00%	0	0	
chr18	56,870,314	56,871,877	1,563	3.13%	1563	100.00%	0	0.00%	0	0.00%	0	0	
chr18	59,394,743	59,395,053	310	50.00%	121	39.03%	0	0.00%	0	0.00%	0	0	

chr18	59,706,961	59,707,481	520	25.00%	0	0.00%	0	0.00%	0	0.00%	0	0	
chr18	65,763,514	65,763,786	272	50.00%	267	98.16%	0	0.00%	0	0.00%	0	0	
chr18	72,816,838	72,817,202	364	3.13%	0	0.00%	0	0.00%	0	0.00%	0	0	
chr18	75,084,721	75,085,063	342	12.50%	0	0.00%	0	0.00%	0	0.00%	0	0	
chr18	76,413,015	76,413,241	226	25.00%	0	0.00%	0	0.00%	0	0.00%	0	0	
chr18	76,701,020	76,701,256	236	50.00%	236	100.00%	0	0.00%	0	0.00%	0	0	
chr19	427,597	427,909	312	46.88%	0	0.00%	0	0.00%	0	0.00%	312	0	*SHC2*
chr19	786,390	786,690	300	18.75%	131	43.67%	0	0.00%	0	0.00%	0	0	
chr19	866,559	866,923	364	31.25%	167	45.88%	0	0.00%	0	0.00%	0	0	
chr19	2,128,753	2,129,189	436	46.88%	177	40.60%	0	0.00%	0	0.00%	436	74	*AP3D1*
chr19	2,559,640	2,580,395	20,755	3.13%	13564	65.35%	0	0.00%	0	0.00%	20755	0	*GNG7*
chr19	4,798,029	4,798,393	364	6.25%	344	94.51%	0	0.00%	0	0.00%	0	0	
chr19	4,937,517	4,940,491	2,974	3.13%	2599	87.39%	0	0.00%	0	0.00%	2974	0	*UHRF1*
chr19	6,657,121	6,657,437	316	3.13%	316	100.00%	316	100.00%	316	100.00%	0	0	
chr19	10,082,319	10,082,592	273	18.75%	0	0.00%	0	0.00%	0	0.00%	273	0	*COL5A3*
chr19	10,633,436	10,634,389	953	3.13%	767	80.48%	0	0.00%	0	0.00%	0	0	
chr19	13,295,710	13,295,958	248	3.13%	244	98.39%	0	0.00%	0	0.00%	0	0	
chr19	17,425,746	17,426,007	261	46.88%	240	91.95%	0	0.00%	0	0.00%	261	0	*DDA1*
chr19	20,807,206	20,807,434	228	50.00%	0	0.00%	0	0.00%	0	0.00%	228	228	*ZNF626*
chr19	20,887,454	20,889,931	2,477	3.13%	1281	51.72%	0	0.00%	0	0.00%	0	0	
chr19	21,406,844	21,407,819	975	3.13%	655	67.18%	0	0.00%	0	0.00%	0	0	
chr19	23,811,874	23,828,238	16,364	3.13%	13325	81.43%	0	0.00%	0	0.00%	0	0	
chr19	30,003,467	30,003,724	257	46.88%	244	94.94%	0	0.00%	0	0.00%	0	0	
chr19	30,004,465	30,005,551	1,086	25.00%	1027	94.57%	0	0.00%	0	0.00%	0	0	
chr19	31,495,469	31,495,757	288	25.00%	259	89.93%	0	0.00%	0	0.00%	0	0	
chr19	43,034,527	43,035,932	1,405	3.13%	910	64.77%	0	0.00%	0	0.00%	0	0	
chr19	44,195,639	44,197,972	2,333	3.13%	1172	50.24%	0	0.00%	0	0.00%	0	0	
chr19	51,128,836	51,129,146	310	25.00%	289	93.23%	0	0.00%	0	0.00%	310	0	*SYT3*
chr19	52,666,798	52,667,397	599	3.13%	293	48.91%	0	0.00%	0	0.00%	599	0	*ZNF836*
chr19	55,671,039	55,671,249	210	50.00%	186	88.57%	0	0.00%	0	0.00%	210	0	*C19orf51*
chr19	56,039,030	56,039,231	201	37.50%	201	100.00%	0	0.00%	0	0.00%	0	0	
chr2	235,401	236,209	808	31.25%	0	0.00%	0	0.00%	0	0.00%	808	0	*SH3YL1*
chr2	863,806	864,539	733	46.88%	0	0.00%	0	0.00%	0	0.00%	306	306	*LOC339822*
chr2	1,530,784	1,531,674	890	34.38%	0	0.00%	0	0.00%	0	0.00%	890	0	*TPO*
chr2	1,641,675	1,641,922	247	9.38%	0	0.00%	0	0.00%	0	0.00%	247	0	*PXDN*
chr2	3,245,968	3,246,215	247	50.00%	0	0.00%	0	0.00%	0	0.00%	247	0	*TSSC1*
chr2	9,285,462	9,294,435	8,973	3.13%	3756	41.86%	0	0.00%	0	0.00%	0	0	
chr2	10,373,777	10,374,090	313	15.63%	313	100.00%	0	0.00%	0	0.00%	0	0	
chr2	11,994,348	11,994,686	338	21.88%	0	0.00%	0	0.00%	0	0.00%	0	0	
chr2	22,673,350	22,673,594	244	6.25%	91	37.30%	0	0.00%	0	0.00%	0	0	
chr2	42,832,269	42,833,431	1,162	53.13%	1113	95.78%	0	0.00%	0	0.00%	1162	0	*MTA3*
chr2	48,791,540	48,791,845	305	50.00%	299	98.03%	0	0.00%	0	0.00%	305	0	*STON1*
chr2	52,492,452	52,493,305	853	6.25%	853	100.00%	0	0.00%	0	0.00%	0	0	
chr2	70,205,240	70,213,422	8,182	3.13%	4359	53.28%	0	0.00%	0	0.00%	8182	0	*LOC400960*
chr2	80,964,895	80,965,416	521	3.13%	521	100.00%	0	0.00%	0	0.00%	0	0	
chr2	85,725,374	85,726,885	1,511	3.13%	1493	98.81%	0	0.00%	0	0.00%	0	0	
chr2	88,429,632	88,429,941	309	31.25%	42	13.59%	0	0.00%	0	0.00%	0	0	
chr2	104,479,979	104,480,245	266	3.13%	0	0.00%	0	0.00%	0	0.00%	0	0	
chr2	114,964,693	114,965,012	319	6.25%	319	100.00%	0	0.00%	0	0.00%	0	0	
chr2	116,196,338	116,196,808	470	3.13%	0	0.00%	0	0.00%	0	0.00%	470	0	*DPP10*
chr2	118,795,506	118,802,676	7,170	3.13%	4280	59.69%	0	0.00%	0	0.00%	0	0	
chr2	147,065,680	147,066,811	1,131	3.13%	1131	100.00%	0	0.00%	0	0.00%	0	0	
chr2	163,608,021	163,608,331	310	53.13%	108	34.84%	0	0.00%	0	0.00%	310	0	*KCNH7*
chr2	164,004,438	164,009,357	4,919	3.13%	2344	47.65%	0	0.00%	0	0.00%	0	0	
chr2	164,106,927	164,108,843	1,916	3.13%	242	12.63%	0	0.00%	0	0.00%	0	0	
chr2	205,252,312	205,252,575	263	3.13%	5	1.90%	0	0.00%	0	0.00%	0	0	
chr2	218,800,592	218,801,775	1,183	3.13%	376	31.78%	0	0.00%	0	0.00%	1183	0	*TNS1*
chr2	228,642,439	228,642,725	286	50.00%	286	100.00%	0	0.00%	0	0.00%	0	0	
chr2	240,652,100	240,652,472	372	3.13%	186	50.00%	0	0.00%	0	0.00%	0	0	
chr2	242,680,952	242,681,234	282	12.50%	0	0.00%	0	0.00%	0	0.00%	282	0	*D2HGDH*
chr2	242,744,337	242,744,663	326	50.00%	0	0.00%	0	0.00%	0	0.00%	0	0	
chr20	1,820,332	1,820,559	227	40.63%	6	2.64%	0	0.00%	0	0.00%	0	0	
chr20	2,878,250	2,878,965	715	50.00%	715	100.00%	0	0.00%	0	0.00%	715	0	*PTPRA*
chr20	3,734,572	3,735,043	471	3.13%	0	0.00%	0	0.00%	0	0.00%	471	233	*C20orf27*
chr20	3,734,721	3,736,110	1,389	3.13%	0	0.00%	0	0.00%	0	0.00%	1389	207	*C20orf27*
chr20	14,683,179	14,683,398	219	50.00%	219	100.00%	0	0.00%	0	0.00%	219	0	*MACROD2*
chr20	33,274,167	33,275,960	1,793	3.13%	1078	60.12%	0	0.00%	0	0.00%	0	0	
chr20	44,189,949	44,191,058	1,109	3.13%	554	49.95%	0	0.00%	0	0.00%	1109	110	*WFDC8*
chr20	58,111,583	58,113,053	1,470	3.13%	1433	97.48%	0	0.00%	0	0.00%	0	0	
chr21	23,560,465	23,561,522	1,057	3.13%	574	54.30%	0	0.00%	0	0.00%	0	0	
chr21	24,469,143	24,471,043	1,900	3.13%	729	38.37%	0	0.00%	0	0.00%	0	0	
chr21	41,346,159	41,346,467	308	9.38%	204	66.23%	0	0.00%	0	0.00%	0	0	
chr21	44,328,641	44,328,878	237	3.13%	0	0.00%	0	0.00%	0	0.00%	237	0	*NDUFV3*
chr21	46,927,046	46,927,411	365	21.88%	0	0.00%	0	0.00%	0	0.00%	365	0	*COL18A1*
chr22	33,575,773	33,575,993	220	3.13%	178	80.91%	0	0.00%	0	0.00%	0	0	
chr22	37,560,420	37,561,044	624	6.25%	135	21.63%	0	0.00%	0	0.00%	0	0	
chr22	43,872,578	43,873,621	1,043	12.50%	154	14.77%	0	0.00%	0	0.00%	1043	0	*MPPED1*
chr22	48,778,994	48,787,383	8,389	3.13%	7121	84.89%	0	0.00%	0	0.00%	0	0	
chr3	1,805,844	1,806,145	301	9.38%	278	92.36%	0	0.00%	0	0.00%	0	0	
chr3	1,805,893	1,806,125	232	9.38%	209	90.09%	0	0.00%	0	0.00%	0	0	
chr3	7,013,569	7,015,349	1,780	3.13%	1096	61.57%	0	0.00%	0	0.00%	1780	0	*GRM7*
chr3	7,530,660	7,585,722	55,062	3.13%	29296	53.21%	0	0.00%	0	0.00%	55062	0	*GRM7*
chr3	8,463,968	8,474,714	10,746	3.13%	5425	50.48%	0	0.00%	0	0.00%	10746	0	*LOC100288428*
chr3	8,720,651	8,720,979	328	46.88%	0	0.00%	328	100.00%	328	100.00%	0	0	
chr3	9,878,636	9,878,899	263	50.00%	228	86.69%	0	0.00%	0	0.00%	0	0	
chr3	19,776,724	19,777,648	924	3.13%	468	50.65%	0	0.00%	0	0.00%	0	0	
chr3	28,463,041	28,472,748	9,707	3.13%	5310	54.70%	0	0.00%	0	0.00%	9707	0	*ZCWPW2*
chr3	32,667,645	32,667,893	248	18.75%	248	100.00%	0	0.00%	0	0.00%	0	0	
chr3	34,727,758	34,808,698	80,940	3.13%	51383	63.48%	0	0.00%	0	0.00%	0	0	
chr3	36,396,278	36,416,915	20,637	3.13%	17231	83.50%	0	0.00%	0	0.00%	0	0	
chr3	41,423,395	41,424,247	852	3.13%	428	50.23%	0	0.00%	0	0.00%	852	0	*ULK4*
chr3	52,625,746	52,626,049	303	50.00%	303	100.00%	0	0.00%	0	0.00%	303	0	*PBRM1*

chr3	58,149,680	58,150,499	819	18.75%	164	20.02%	0	0.00%	0	0.00%	819	0	*FLNB*
chr3	75,090,458	75,090,867	409	3.13%	409	100.00%	0	0.00%	0	0.00%	0	0	
chr3	86,882,734	86,883,111	377	50.00%	196	51.99%	0	0.00%	0	0.00%	0	0	
chr3	100,188,797	100,194,221	5,424	3.13%	5377	99.13%	0	0.00%	0	0.00%	0	0	
chr3	108,158,822	108,161,101	2,279	3.13%	303	13.30%	0	0.00%	0	0.00%	2279	243	*MYH15*
chr3	117,256,891	117,257,490	599	6.25%	85	14.19%	0	0.00%	0	0.00%	0	0	
chr3	122,196,823	122,197,399	576	3.13%	551	95.66%	0	0.00%	0	0.00%	576	0	*KPNA1*
chr3	128,173,016	128,173,288	272	25.00%	0	0.00%	0	0.00%	0	0.00%	0	0	
chr3	128,745,128	128,753,910	8,782	3.13%	4835	55.06%	0	0.00%	0	0.00%	8782	469	*CCDC48*
chr3	130,288,858	130,302,136	13,278	3.13%	3843	28.94%	0	0.00%	0	0.00%	13278	1569	*COL6A6*
chr3	142,081,390	142,081,856	466	50.00%	421	90.34%	0	0.00%	0	0.00%	466	0	*XRN1*
chr3	144,693,113	144,693,362	249	50.00%	29	11.65%	0	0.00%	0	0.00%	0	0	
chr3	147,775,968	147,779,958	3,990	6.25%	2184	54.74%	0	0.00%	0	0.00%	0	0	
chr3	148,516,259	148,520,666	4,407	3.13%	2183	49.53%	0	0.00%	0	0.00%	0	0	
chr3	179,835,746	179,835,963	217	12.50%	0	0.00%	0	0.00%	0	0.00%	0	0	
chr3	184,470,887	184,471,195	308	68.75%	239	77.60%	0	0.00%	0	0.00%	0	0	
chr3	191,653,520	191,666,153	12,633	3.13%	5811	46.00%	0	0.00%	0	0.00%	0	0	
chr3	195,607,293	195,607,847	554	50.00%	0	0.00%	0	0.00%	0	0.00%	554	0	*TNK2*
chr3	197,320,315	197,322,131	1,816	3.13%	1134	62.44%	0	0.00%	0	0.00%	0	0	
chr4	912,641	912,873	232	43.75%	0	0.00%	0	0.00%	0	0.00%	232	0	*GAK*
chr4	3,310,670	3,311,346	676	12.50%	0	0.00%	0	0.00%	0	0.00%	0	0	
chr4	3,492,426	3,492,919	493	46.88%	384	77.89%	0	0.00%	0	0.00%	493	0	*DOK7*
chr4	6,020,021	6,021,083	1,062	3.13%	177	16.67%	0	0.00%	0	0.00%	0	0	
chr4	7,167,186	7,167,388	202	12.50%	0	0.00%	0	0.00%	0	0.00%	0	0	
chr4	11,238,570	11,244,155	5,585	3.13%	5247	93.95%	0	0.00%	0	0.00%	0	0	
chr4	24,022,514	24,026,993	4,479	3.13%	694	15.49%	0	0.00%	0	0.00%	0	0	
chr4	45,533,521	45,534,433	912	3.13%	896	98.25%	0	0.00%	0	0.00%	0	0	
chr4	53,403,755	53,425,527	21,772	3.13%	13544	62.21%	0	0.00%	0	0.00%	0	0	
chr4	53,435,839	53,440,663	4,824	3.13%	2574	53.36%	0	0.00%	0	0.00%	0	0	
chr4	72,983,547	72,983,787	240	3.13%	44	18.33%	0	0.00%	0	0.00%	240	0	*NPFFR2*
chr4	77,501,775	77,506,894	5,119	3.13%	1417	27.68%	0	0.00%	0	0.00%	5119	0	*SHROOM3*
chr4	81,586,405	81,591,565	5,160	3.13%	3818	73.99%	0	0.00%	0	0.00%	5160	0	*C4orf22*
chr4	95,787,662	95,787,922	260	3.13%	198	76.15%	0	0.00%	0	0.00%	260	0	*BMPR1B*
chr4	104,139,341	104,147,090	7,749	3.13%	7749	100.00%	0	0.00%	0	0.00%	0	0	
chr4	107,374,172	107,375,120	948	3.13%	16	1.69%	0	0.00%	0	0.00%	0	0	
chr4	108,228,581	108,229,041	460	12.50%	448	97.39%	0	0.00%	0	0.00%	0	0	
chr4	111,572,798	111,575,193	2,395	3.13%	662	27.64%	0	0.00%	0	0.00%	0	0	
chr4	125,996,873	126,006,183	9,310	3.13%	1857	19.95%	0	0.00%	0	0.00%	0	0	
chr4	150,438,022	150,440,465	2,443	3.13%	1338	54.77%	0	0.00%	0	0.00%	0	0	
chr4	154,165,243	154,165,595	352	46.88%	341	96.88%	0	0.00%	0	0.00%	352	0	*TRIM2*
chr4	165,922,956	165,923,515	559	21.88%	559	100.00%	0	0.00%	0	0.00%	0	0	
chr4	171,022,639	171,023,403	764	3.13%	764	100.00%	0	0.00%	0	0.00%	0	0	
chr4	188,685,511	188,685,897	386	46.88%	380	98.45%	0	0.00%	0	0.00%	0	0	
chr4	188,762,349	188,762,705	356	3.13%	0	0.00%	0	0.00%	0	0.00%	0	0	
chr4	188,991,734	188,992,059	325	3.13%	315	96.92%	0	0.00%	0	0.00%	0	0	
chr4	189,792,111	189,792,317	206	6.25%	0	0.00%	0	0.00%	0	0.00%	0	0	
chr4	190,417,545	190,417,888	343	31.25%	0	0.00%	0	0.00%	0	0.00%	0	0	
chr5	53,073	53,565	492	50.00%	0	0.00%	0	0.00%	0	0.00%	0	0	
chr5	164,421	165,351	930	21.88%	185	19.89%	0	0.00%	0	0.00%	930	0	*PLEKHG4B*
chr5	467,565	468,072	507	62.50%	0	0.00%	0	0.00%	0	0.00%	0	0	
chr5	474,802	475,159	357	18.75%	151	42.30%	0	0.00%	0	0.00%	357	162	*SLC9A3*
chr5	2,152,472	2,152,678	206	15.63%	0	0.00%	0	0.00%	0	0.00%	0	0	
chr5	15,575,003	15,575,404	401	3.13%	205	51.12%	0	0.00%	0	0.00%	401	0	*FBXL7*
chr5	16,944,175	16,944,433	258	15.63%	250	96.90%	0	0.00%	0	0.00%	0	0	
chr5	19,849,635	19,849,893	258	50.00%	168	65.12%	0	0.00%	0	0.00%	258	0	*CDH18*
chr5	23,060,410	23,060,622	212	9.38%	32	15.09%	0	0.00%	0	0.00%	0	0	
chr5	29,868,937	29,869,143	206	25.00%	206	100.00%	0	0.00%	0	0.00%	0	0	
chr5	32,713,650	32,714,100	450	15.63%	0	0.00%	0	0.00%	0	0.00%	450	0	*NPR3*
chr5	37,784,672	37,784,926	254	28.13%	254	100.00%	0	0.00%	0	0.00%	0	0	
chr5	50,884,632	50,885,031	399	53.13%	25	6.27%	0	0.00%	0	0.00%	0	0	
chr5	53,096,916	53,100,331	3,415	3.13%	3415	100.00%	0	0.00%	0	0.00%	0	0	
chr5	70,847,607	70,847,891	284	46.88%	284	100.00%	0	0.00%	0	0.00%	284	0	*BDP1*
chr5	73,567,482	73,567,699	217	6.25%	132	60.83%	0	0.00%	0	0.00%	0	0	
chr5	90,854,961	90,855,255	294	3.13%	294	100.00%	0	0.00%	0	0.00%	0	0	
chr5	91,409,606	91,409,850	244	65.63%	114	46.72%	0	0.00%	0	0.00%	0	0	
chr5	98,587,142	98,592,586	5,444	3.13%	5444	100.00%	0	0.00%	0	0.00%	0	0	
chr5	109,359,488	109,380,442	20,954	3.13%	12444	59.39%	0	0.00%	0	0.00%	0	0	
chr5	117,449,094	117,449,339	245	12.50%	227	92.65%	0	0.00%	0	0.00%	0	0	
chr5	120,727,289	120,727,574	285	50.00%	32	11.23%	0	0.00%	0	0.00%	0	0	
chr5	128,327,860	128,328,121	261	50.00%	0	0.00%	0	0.00%	0	0.00%	261	0	*SLC27A6*
chr5	137,820,055	137,830,443	10,388	3.13%	4487	43.19%	0	0.00%	0	0.00%	0	0	
chr5	143,660,386	143,660,648	262	3.13%	262	100.00%	0	0.00%	0	0.00%	262	0	*KCTD16*
chr5	147,359,451	147,362,997	3,546	3.13%	1020	28.76%	0	0.00%	0	0.00%	0	0	
chr5	149,049,867	149,050,257	390	50.00%	390	100.00%	0	0.00%	0	0.00%	0	0	
chr5	164,065,114	164,070,943	5,829	3.13%	2150	36.88%	0	0.00%	0	0.00%	0	0	
chr5	175,957,293	175,957,606	313	43.75%	0	0.00%	0	0.00%	0	0.00%	313	24	*RNF44*
chr5	177,389,476	177,401,297	11,821	3.13%	6566	55.55%	1641	13.88%	0	0.00%	0	0	
chr5	177,412,870	177,413,166	296	50.00%	118	39.86%	0	0.00%	0	0.00%	0	0	
chr5	179,077,368	179,077,644	276	53.13%	276	100.00%	276	100.00%	276	100.00%	0	0	
chr5	179,100,319	179,113,985	13,666	3.13%	10057	73.59%	0	0.00%	0	0.00%	2417	810	*CBY3*
chr6	1,441,540	1,441,834	294	46.88%	16	5.44%	0	0.00%	0	0.00%	0	0	
chr6	2,468,602	2,468,854	252	3.13%	231	91.67%	0	0.00%	0	0.00%	0	0	
chr6	32,661,656	32,661,955	299	50.00%	299	100.00%	0	0.00%	0	0.00%	0	0	
chr6	34,987,706	34,987,930	224	3.13%	0	0.00%	0	0.00%	0	0.00%	224	0	*ANKS1A*
chr6	41,539,065	41,539,802	737	3.13%	47	6.38%	0	0.00%	0	0.00%	737	0	*FOXP4*
chr6	49,646,510	49,655,659	9,149	3.13%	6133	67.03%	0	0.00%	0	0.00%	0	0	
chr6	65,356,925	65,357,739	814	3.13%	108	13.27%	0	0.00%	0	0.00%	814	0	*EYS*
chr6	72,025,963	72,026,252	289	15.63%	283	97.92%	0	0.00%	0	0.00%	0	0	
chr6	72,355,168	72,355,375	207	21.88%	199	96.14%	0	0.00%	0	0.00%	0	0	
chr6	78,427,702	78,429,266	1,564	46.88%	1564	100.00%	0	0.00%	0	0.00%	0	0	
chr6	95,400,702	95,400,928	226	46.88%	226	100.00%	0	0.00%	0	0.00%	0	0	
chr6	102,741,445	102,741,692	247	43.75%	247	100.00%	0	0.00%	0	0.00%	0	0	

chr6	128,573,064	128,576,726	3,662	3.13%	376	10.27%	0	0.00%	0	0.00%	3662	0	*PTPRK*
chr6	128,639,190	128,642,167	2,977	3.13%	365	12.26%	0	0.00%	0	0.00%	2977	0	*PTPRK*
chr6	132,730,142	132,730,862	720	3.13%	224	31.11%	0	0.00%	0	0.00%	0	0	
chr6	133,667,835	133,668,120	285	3.13%	144	50.53%	0	0.00%	0	0.00%	285	0	*EYA4*
chr6	135,552,060	135,552,268	208	3.13%	0	0.00%	0	0.00%	0	0.00%	0	0	
chr6	146,771,778	146,777,541	5,763	3.13%	5760	99.95%	5763	100.00%	5763	100.00%	0	0	
chr6	152,053,376	152,060,637	7,261	3.13%	2724	37.52%	0	0.00%	0	0.00%	7261	0	*ESR1*
chr6	159,181,358	159,181,776	418	3.13%	0	0.00%	0	0.00%	0	0.00%	418	105	*SYTL3*
chr6	161,511,567	161,511,784	217	50.00%	193	88.94%	0	0.00%	0	0.00%	217	0	*MAP3K4*
chr6	164,422,247	164,424,546	2,299	3.13%	95	4.13%	0	0.00%	0	0.00%	0	0	
chr6	168,968,585	168,968,857	272	3.13%	0	0.00%	0	0.00%	0	0.00%	272	0	*SMOC2*
chr6	169,462,657	169,462,869	212	15.63%	0	0.00%	0	0.00%	0	0.00%	0	0	
chr6	170,208,259	170,208,493	234	90.63%	0	0.00%	0	0.00%	0	0.00%	0	0	
chr6	170,208,974	170,209,459	485	37.50%	177	36.49%	0	0.00%	0	0.00%	0	0	
chr7	102,886	103,235	349	43.75%	69	19.77%	0	0.00%	0	0.00%	0	0	
chr7	224,045	224,319	274	43.75%	0	0.00%	0	0.00%	0	0.00%	274	0	*FAM20C*
chr7	303,811	304,046	235	6.25%	0	0.00%	0	0.00%	0	0.00%	0	0	
chr7	337,032	337,338	306	15.63%	0	0.00%	0	0.00%	0	0.00%	0	0	
chr7	438,531	438,783	252	3.13%	32	12.70%	0	0.00%	0	0.00%	0	0	
chr7	497,030	497,523	493	9.38%	0	0.00%	0	0.00%	0	0.00%	0	0	
chr7	571,160	571,431	271	50.00%	222	81.92%	0	0.00%	0	0.00%	0	0	
chr7	648,281	648,508	227	46.88%	0	0.00%	0	0.00%	0	0.00%	227	0	*PRKAR1B*
chr7	2,417,083	2,417,372	289	37.50%	0	0.00%	0	0.00%	0	0.00%	289	0	*EIF3B*
chr7	2,417,967	2,418,183	216	43.75%	0	0.00%	0	0.00%	0	0.00%	216	0	*EIF3B*
chr7	4,219,857	4,220,128	271	28.13%	257	94.83%	0	0.00%	0	0.00%	271	0	*SDK1*
chr7	14,930,380	14,932,244	1,864	3.13%	233	12.50%	0	0.00%	0	0.00%	0	0	
chr7	23,944,023	23,944,223	200	100.00%	200	100.00%	0	0.00%	0	0.00%	0	0	
chr7	26,216,313	26,216,756	443	6.25%	443	100.00%	0	0.00%	0	0.00%	443	0	*NFE2L3*
chr7	27,935,795	27,936,005	210	3.13%	0	0.00%	0	0.00%	0	0.00%	210	0	*JAZF1*
chr7	29,441,733	29,446,277	4,544	3.13%	991	21.81%	0	0.00%	0	0.00%	4544	0	*CHN2*
chr7	32,550,193	32,550,401	208	3.13%	0	0.00%	0	0.00%	0	0.00%	208	0	*AVL9*
chr7	55,959,042	55,959,540	498	3.13%	243	48.80%	0	0.00%	0	0.00%	0	0	
chr7	66,140,328	66,143,568	3,240	3.13%	2891	89.23%	0	0.00%	0	0.00%	0	0	
chr7	91,888,096	91,889,198	1,102	3.13%	629	57.08%	0	0.00%	0	0.00%	1102	0	*ANKIB1*
chr7	109,693,594	109,693,807	213	37.50%	211	99.06%	0	0.00%	0	0.00%	0	0	
chr7	121,722,813	121,723,737	924	3.13%	288	31.17%	0	0.00%	0	0.00%	924	0	*AASS*
chr7	124,299,828	124,300,161	333	50.00%	308	92.49%	0	0.00%	0	0.00%	0	0	
chr7	125,211,625	125,215,356	3,731	3.13%	763	20.45%	0	0.00%	0	0.00%	0	0	
chr7	136,447,990	136,457,896	9,906	3.13%	8745	88.28%	0	0.00%	0	0.00%	0	0	
chr7	147,848,252	147,850,146	1,894	3.13%	1362	71.91%	0	0.00%	0	0.00%	1894	0	*MIR548T*
chr7	151,591,848	151,592,112	264	15.63%	246	93.18%	0	0.00%	0	0.00%	0	0	
chr7	157,293,661	157,294,002	341	12.50%	0	0.00%	0	0.00%	0	0.00%	0	0	
chr7	157,721,966	157,722,576	610	3.13%	0	0.00%	0	0.00%	0	0.00%	610	0	*PTPRN2*
chr7	157,747,189	157,747,493	304	50.00%	109	35.86%	0	0.00%	0	0.00%	304	0	*PTPRN2*
chr7	158,621,448	158,621,670	222	46.88%	23	10.36%	0	0.00%	0	0.00%	222	0	*ESYT2*
chr7	158,790,466	158,790,862	396	25.00%	0	0.00%	0	0.00%	0	0.00%	0	0	
chr8	645,517	646,591	1,074	28.13%	1022	95.16%	0	0.00%	0	0.00%	1074	0	*ERICH1*
chr8	735,581	735,813	232	34.38%	0	0.00%	0	0.00%	0	0.00%	0	0	
chr8	1,409,190	1,409,726	536	31.25%	0	0.00%	0	0.00%	0	0.00%	0	0	
chr8	1,776,581	1,776,963	382	6.25%	0	0.00%	0	0.00%	0	0.00%	382	0	*ARHGEF10*
chr8	23,933,950	23,934,174	224	50.00%	224	100.00%	0	0.00%	0	0.00%	0	0	
chr8	34,802,883	34,840,424	37,541	3.13%	14005	37.31%	0	0.00%	0	0.00%	0	0	
chr8	37,703,764	37,704,131	367	3.13%	255	69.48%	0	0.00%	0	0.00%	367	0	*BRF2*
chr8	48,646,056	48,647,227	1,171	6.25%	367	31.34%	0	0.00%	0	0.00%	1171	0	*KIAA0146*
chr8	76,934,324	76,935,468	1,144	3.13%	276	24.13%	0	0.00%	0	0.00%	0	0	
chr8	79,079,338	79,079,586	248	9.38%	248	100.00%	0	0.00%	0	0.00%	0	0	
chr8	105,267,475	105,267,693	218	9.38%	0	0.00%	0	0.00%	0	0.00%	0	0	
chr8	120,510,323	120,510,566	243	50.00%	208	85.60%	0	0.00%	0	0.00%	0	0	
chr8	129,253,137	129,253,361	224	3.13%	194	86.61%	0	0.00%	0	0.00%	0	0	
chr8	134,359,494	134,360,047	553	3.13%	546	98.73%	0	0.00%	0	0.00%	0	0	
chr8	140,422,276	140,422,583	307	25.00%	307	100.00%	0	0.00%	0	0.00%	0	0	
chr8	142,507,125	142,507,370	245	21.88%	238	97.14%	0	0.00%	0	0.00%	245	0	*FLJ43860*
chr8	143,132,737	143,133,768	1,031	3.13%	1031	100.00%	0	0.00%	0	0.00%	0	0	
chr8	143,308,379	143,308,618	239	12.50%	0	0.00%	0	0.00%	0	0.00%	239	0	*TSNARE1*
chr8	144,774,640	144,775,006	366	18.75%	0	0.00%	0	0.00%	0	0.00%	366	0	*ZNF707*
chr8	145,503,128	145,503,373	245	37.50%	0	0.00%	0	0.00%	0	0.00%	245	0	*BOP1*
chr9	12,086,778	12,118,616	31,838	6.25%	20932	65.75%	0	0.00%	0	0.00%	0	0	
chr9	13,155,011	13,155,243	232	12.50%	232	100.00%	0	0.00%	0	0.00%	232	0	*MPDZ*
chr9	14,498,636	14,498,864	228	50.00%	94	41.23%	0	0.00%	0	0.00%	0	0	
chr9	19,473,868	19,474,099	231	21.88%	231	100.00%	0	0.00%	0	0.00%	0	0	
chr9	34,389,624	34,390,843	1,219	6.25%	1204	98.77%	0	0.00%	0	0.00%	1219	0	*C9orf24*
chr9	74,471,627	74,474,316	2,689	6.25%	699	25.99%	0	0.00%	0	0.00%	0	0	
chr9	74,914,442	74,914,693	251	3.13%	72	28.69%	0	0.00%	0	0.00%	0	0	
chr9	91,517,691	91,526,350	8,659	3.13%	8603	99.35%	0	0.00%	0	0.00%	0	0	
chr9	98,850,174	98,851,178	1,004	6.25%	432	43.03%	0	0.00%	0	0.00%	1004	0	*LOC158435*
chr9	116,662,961	116,663,389	428	12.50%	0	0.00%	0	0.00%	0	0.00%	428	0	*ZNF618*
chr9	117,890,601	117,892,373	1,772	3.13%	1282	72.35%	0	0.00%	0	0.00%	0	0	
chr9	119,196,282	119,268,516	72,234	3.13%	35962	49.79%	0	0.00%	0	0.00%	72234	489	*ASTN2*
chr9	130,232,065	130,234,006	1,941	3.13%	1621	83.51%	0	0.00%	0	0.00%	1941	0	*LRSAM1*
chr9	130,900,646	130,900,986	340	15.63%	0	0.00%	340	100.00%	340	100.00%	0	0	
chr9	131,067,958	131,069,015	1,057	3.13%	606	57.33%	0	0.00%	0	0.00%	0	0	
chr9	131,203,307	131,205,024	1,717	3.13%	1395	81.25%	0	0.00%	0	0.00%	0	0	
chr9	136,638,043	136,638,281	238	15.63%	0	0.00%	0	0.00%	0	0.00%	238	0	*VAV2*
chr9	136,975,359	136,975,697	338	3.13%	338	100.00%	0	0.00%	0	0.00%	0	0	
chr9	137,099,214	137,099,656	442	40.63%	428	96.83%	0	0.00%	0	0.00%	0	0	
chr9	137,684,595	137,684,869	274	40.63%	177	64.60%	0	0.00%	0	0.00%	274	0	*COL5A1*
chr9	138,153,854	138,154,110	256	12.50%	255	99.61%	0	0.00%	0	0.00%	0	0	
chr9	139,492,902	139,493,203	301	12.50%	90	29.90%	0	0.00%	0	0.00%	0	0	
chr9	139,612,580	139,612,828	248	43.75%	0	0.00%	0	0.00%	0	0.00%	248	0	*FAM69B*
chrX	5,304,870	5,312,288	7,418	34.38%	4499	60.65%	0	0.00%	0	0.00%	0	0	
chrX	5,913,077	5,913,341	264	34.38%	0	0.00%	0	0.00%	0	0.00%	264	0	*NLGN4X*
chrX	11,354,128	11,357,507	3,379	34.38%	1300	38.47%	0	0.00%	0	0.00%	3379	0	*ARHGAP6*

chrX	23,979,262	23,979,551	289	31.25%	287	99.31%	0	0.00%	0	0.00%	0	0	
chrX	24,598,828	24,600,221	1,393	34.38%	736	52.84%	0	0.00%	0	0.00%	1393	0	*PCYT1B*
chrX	31,085,573	31,085,802	229	31.25%	223	97.38%	0	0.00%	0	0.00%	0	0	
chrX	36,389,607	36,389,988	381	40.63%	372	97.64%	0	0.00%	0	0.00%	381	0	*CXorf30*
chrX	36,903,399	36,903,624	225	34.38%	225	100.00%	0	0.00%	0	0.00%	0	0	
chrX	41,214,842	41,215,402	560	40.63%	558	99.64%	0	0.00%	0	0.00%	0	0	
chrX	95,603,304	95,605,094	1,790	34.38%	1790	100.00%	0	0.00%	0	0.00%	0	0	
chrX	96,606,699	96,608,740	2,041	34.38%	669	32.78%	0	0.00%	0	0.00%	2041	0	*DIAPH2*
chrX	143,750,815	143,751,072	257	34.38%	224	87.16%	0	0.00%	0	0.00%	0	0	
chrX	145,614,475	145,649,529	35,054	34.38%	19954	56.92%	0	0.00%	0	0.00%	0	0	
chrX	146,684,469	146,684,726	257	43.75%	251	97.67%	0	0.00%	0	0.00%	0	0	
chrX	151,320,588	151,363,997	43,409	31.25%	24807	57.15%	0	0.00%	0	0.00%	28364	1614	*GABRA3*
chrY	8,674,461	8,674,682	221	71.88%	221	100.00%	0	0.00%	0	0.00%	221	0	*TTTY11*
chrY	21,560,649	21,561,839	1,190	71.88%	1190	100.00%	0	0.00%	0	0.00%	0	0	
chrY	22,224,814	22,231,598	6,784	43.75%	6784	100.00%	6186	91.19%	6186	91.19%	0	0	
chrY	22,255,556	22,262,713	7,157	37.50%	7097	99.16%	7157	100.00%	7157	100.00%	0	0	
chrY	22,269,469	22,274,269	4,800	37.50%	4784	99.67%	3800	79.17%	3800	79.17%	0	0	
chrY	22,273,488	22,282,041	8,553	68.75%	8537	99.81%	8553	100.00%	8553	100.00%	0	0	
chrY	22,278,620	22,279,262	642	68.75%	642	100.00%	642	100.00%	642	100.00%	0	0	
chrY	22,279,753	22,282,014	2,261	68.75%	2261	100.00%	2261	100.00%	2261	100.00%	0	0	
chrY	22,314,683	22,316,163	1,480	71.88%	1452	98.11%	1480	100.00%	1480	100.00%	0	0	
chrY	22,322,260	22,325,521	3,261	90.63%	3250	99.66%	3261	100.00%	3261	100.00%	0	0	
chrY	22,351,602	22,355,881	4,279	75.00%	4254	99.42%	4279	100.00%	4279	100.00%	0	0	
chrY	22,351,906	22,355,043	3,137	75.00%	3137	100.00%	3137	100.00%	3137	100.00%	0	0	
chrY	22,359,564	22,361,369	1,805	75.00%	1805	100.00%	1805	100.00%	1805	100.00%	0	0	
chrY	22,359,820	22,361,869	2,049	81.25%	2049	100.00%	2049	100.00%	2049	100.00%	0	0	
chrY	22,364,051	22,368,307	4,256	75.00%	4256	100.00%	4256	100.00%	4256	100.00%	0	0	
chrY	22,435,419	22,436,793	1,374	53.13%	1357	98.76%	1374	100.00%	1374	100.00%	0	0	
chrY	22,455,840	22,471,506	15,666	53.13%	15654	99.92%	6160	39.32%	6160	39.32%	0	0	
chrY	22,460,586	22,463,228	2,642	62.50%	2642	100.00%	1414	53.52%	1414	53.52%	0	0	
chrY	22,484,110	22,494,238	10,128	50.00%	10067	99.40%	10128	100.00%	10128	100.00%	0	0	
chrY	22,496,746	22,507,695	10,949	68.75%	10933	99.85%	10949	100.00%	10949	100.00%	0	0	
chrY	22,507,561	22,508,431	870	43.75%	862	99.08%	870	100.00%	870	100.00%	0	0	
chrY	22,508,587	22,509,115	528	40.63%	528	100.00%	528	100.00%	528	100.00%	0	0	
chrY	22,508,721	22,509,151	430	37.50%	430	100.00%	430	100.00%	430	100.00%	0	0	
chrY	22,511,232	22,511,986	754	37.50%	754	100.00%	754	100.00%	754	100.00%	0	0	

SD: Segmental duplications. WSSD: Whole-genome shotgun sequence detection (duplications detected in the Celera dataset)
